# Energetic and carbon allocation strategies shape coral holobiont responses to combined thermal and nutrient stress

**DOI:** 10.3389/fmicb.2026.1909606

**Published:** 2026-08-10

**Authors:** Kiara Lange, Cécile Rottier, Julie Davenet, Renaud Grover, Christine Ferrier-Pagès

**Affiliations:** Coral Biology Team, Centre Scientifique de Monaco, Monaco, Monaco

**Keywords:** carbon budget, energy reserves, eutrophication, nitrate–phosphate enrichment, octocoral, post-stress recovery, scleractinian, translocation

## Abstract

Climate change and coastal eutrophication increasingly threaten coral reefs, yet their combined impact on coral holobionts remains poorly understood. This study examined the physiological response and carbon budget of two holobionts (*Galaxea fascicularis* in symbiosis with *Cladocopium*; *Heteroxenia fuscescens* in symbiosis with *Durusdinium*) exposed to nitrate–phosphate enrichment and thermal stress (30 °C). In *G. fascicularis*, individual stressors severely reduced photosynthate translocation (−90%) due to a significant increase in symbiont respiration, and this species suffered significant bleaching (86% symbiont loss) after the heat-stress phase. Conversely, *H. fuscescens* was resilient to individual stressors, showing no bleaching and increased carbon translocation under nutrient enrichment or thermal stress alone. Both species exhibited an “energy saving” response following heat stress exposure, significantly increasing lipid and carbohydrate stores. Combined stressors temporarily boosted photosynthetic rates and carbon translocation in *G. fascicularis*, before a collapse in these parameters after the stress. In contrast, under combined stress *H. fuscescens* suffered severe bleaching but maintained high rates of carbon translocation to the host and accumulated substantial energy reserves. These findings suggest that different strategies in carbon allocation dictate competitive success under environmental stress: while *G. fascicularis* prioritizes symbiont maintenance, *H. fuscescens* maintains or enhances translocation to preserve host metabolism. This study highlights the importance of assessing holobiont carbon budgets and energy reserves to predict coral resilience in a changing ocean.

## 1 Introduction

Symbiosis, defined as the close and long-term interaction between organisms of different species, is a fundamental biological phenomenon that shapes the structure, function, and evolution of life on Earth (Thrall et al., 2007). Such interactions influence how organisms acquire resources, adapt to their environments, and persist under changing conditions. Among the different forms of symbiosis, mutualistic relationships, in which both partners benefit, are of particular interest (Bronstein, 2015). In mutualistic symbioses, each organism provides resources or services that enhance the survival, growth, or reproductive success of the other, such as the exchange of nutrients, protection from predators and environmental stress, or assistance with physiological processes like digestion and reproduction. One of the most well-known examples of marine mutualism is the symbiotic relationship between reef-building corals and dinoflagellates of the family Symbiodiniaceae (Davy et al., 2012; LaJeunesse et al., 2018). Symbiodiniaceae supply up to 90% of the host’s energetic requirements through the translocation of photosynthetically fixed carbon (e.g., Muscatine et al., 1981; Tremblay et al., 2014). In addition to carbon fixation, these symbionts are highly efficient at assimilating dissolved inorganic nitrogen (DIN; ammonium, nitrate, nitrite) and phosphorus (DIP; phosphate) from seawater, tightly coupling coral nutrition to ambient nutrient availability (Muscatine et al., 1979; Grover et al., 2002, 2003; Pernice et al., 2012; Ferrier-Pagès et al., 2016). Such symbiosis allows corals to thrive in oligotrophic waters where dissolved inorganic nutrient concentrations are naturally low (D’Elia, 1977; Muscatine et al., 1979; Sorokin, 1993; Kleypas et al., 1999), and to build one of the most biologically diverse ecosystems on Earth (Knowlton et al., 2010).

However, the stability of this symbiosis is highly sensitive to environmental conditions, particularly temperature and nutrient availability. Elevated sea surface temperatures can disrupt the photosynthetic machinery of the Symbiodiniaceae, leading to the production of harmful reactive oxygen species (ROS) (Weis, 2008). To protect themselves from cellular damage, corals may expel their symbiotic algae, resulting in coral bleaching and a severe reduction in energy supply (Hoegh-Guldberg, 1999; Hughes et al., 2017). In addition to ROS production, thermal stress may disrupt the finely tuned carbon exchange between corals and Symbiodiniaceae, for example by reducing translocation of fixed carbon, and creating a carbon deficit for the coral host (Tremblay et al., 2016). Yet, the extent to which different coral–symbiont pairings can maintain carbon translocation under heat stress is unclear, and the mechanisms behind reduced carbon transfer are still debated. It is also not fully known how much carbon is retained by the symbionts for their own survival, or how repeated or chronic thermal stress affects long-term carbon balance. The use of energetic reserves to compensate for the loss of carbon transfer also needs more attention (Grottoli et al., 2014). Addressing these unknowns is crucial for predicting coral resilience in the face of rising ocean temperatures.

In parallel with global warming, coastal eutrophication has emerged as a major local stressor affecting coral reef ecosystems worldwide (Fabricius, 2005, 2011). Nutrient enrichment occurs in many reef regions because of agricultural runoff, wastewater discharge and land-use change, increasing dissolved inorganic nitrogen or phosphorus concentrations above the typically oligotrophic background (DIN, <0.5 μM; DIP, <0.2 μM). Nitrate, the major anthropogenic nitrogen input, is a nitrogen form that is less readily assimilated by corals than ammonium and that can exert deleterious effects (Wiedenmann et al., 2013; Shantz and Burkepile, 2014; Ezzat et al., 2015; Morris et al., 2019). Because of the close nutritional relationship between corals and their symbionts, changes in nutrient availability can therefore strongly influence coral physiology, energy balance, and reef community structure. In scleractinian corals, an imbalanced N:P ratio (too much of one nutrient compared to another one, *sensus* Wiedenmann et al., 2013), is known to reduce calcification and growth rates and impair reproductive output, together with an increased susceptibility to bleaching, disease, and mortality (Koop et al., 2001; Renegar and Riegl, 2005; Wiedenmann et al., 2013; Shantz and Burkepile, 2014; Vega Thurber et al., 2014; Ezzat et al., 2015; Burkepile et al., 2020; Liu et al., 2020; Marangoni et al., 2020; Mezger et al., 2025). However, under moderate and balanced N:P ratios, neutral or even positive effects have been observed, including enhanced symbiont density, photosynthetic performance and growth (Ferrier-Pagès et al., 2000; Koop et al., 2001; Tanaka et al., 2007; Ezzat et al., 2015, 2016b; Dobson et al., 2021). As with heat stress alone, it is however unclear how nutrient availability, particularly nitrogen and phosphorus, affect the proportion of photosynthates transferred from symbionts to host, or whether changes in algal growth and density enhance or reduce carbon translocation over time. Importantly, nutrient effects on corals are strongly modulated by thermal conditions. During heat stress, phosphorus availability, for example, appears critical for sustaining symbiont density, photosynthetic performance, and carbon translocation to the host (Wiedenmann et al., 2013; Ezzat et al., 2016a).

A critical determinant of the corals’ responses to heat and nutrient stress is symbiont identity, which influences not only thermal tolerance but also the stability of cooperative carbon exchange. For example, it has been demonstrated for many host species that symbionts of the genus *Cladocopium* (former clade C; LaJeunesse et al., 2018) may maximize host productivity under ideal, oligotrophic conditions but can shift toward reduced cooperation or even “parasitic” behaviour under thermal or nutrient stress (Stat et al., 2008; Lesser et al., 2013). In contrast, the genus *Durusdinium* (former clade D; LaJeunesse et al., 2018) often provides lower but more resilient carbon fluxes, maintaining mutualistic function across a broader range of environmental perturbations and host species (Rowan, 2004; Stat and Gates, 2011; Cunning et al., 2015). Despite the growing recognition that global warming and local nutrient enrichment interact to shape reef assemblages (Hall et al., 2018; Zhao et al., 2021), comprehensive assessments of the carbon budget of different holobionts under combined thermal and nutrient stress are still lacking, even though carbon acquisition and allocation are central to maintaining metabolic balance and stress resilience.

Here, we address some of these gaps by assessing the responses of a reef-building scleractinian coral (*Galaxea fascicularis*, Linnaeus 1767; hosting *Cladocopium*) and a soft coral from the family Xeniidae (*Heteroxenia fuscescens*, Ehrenberg 1834; hosting *Durusdinium*) to a moderate enrichment in nitrate and phosphate under different temperature conditions. Measurements were performed either under control temperature conditions, or during a short-term heat stress (30 °C for 2 weeks), and “post-stress” phase (return to 25 °C for 2 weeks). We assessed key autotrophic traits, including holobiont carbon budget, *Symbiodiniaceae* density and chlorophyll *a* content, alongside measurements of inorganic nutrient uptake (NH₄^+^, NO₃^−^, PO₄^3−^) and general physiological indicators of holobiont health (oxygen fluxes, energy reserves and tissue C:N:P stoichiometry). We aimed to evaluate (i) the extent to which heat stress or nutrient enrichment affect autotrophic performance and symbiosis stability across the studied coral species; (ii) how do heat stress and nutrient enrichment interact to shape autotrophic performance and symbiosis stability. In other words, we aimed to understand whether the interactive effects of thermal stress and nutrient enrichment are additive, antagonistic, or synergistic across species. By integrating physiological measurements with an explicit assessment of the holobiont carbon budget, this study aims to clarify how ocean warming and inorganic eutrophication differentially affect symbiosis and autotrophic capacity in corals, and how these processes may contribute to ongoing shifts in reef community structure.

## 2 Materials and methods

### 2.1 Biological material

The experiment was conducted on two tropical coral species: the hexacoral *Galaxea fascicularis* (Linnaeus, 1767; order Scleractinia, family Euphylliidae) and the octocoral *Heteroxenia fuscescens* (Ehrenberg, 1834; order Malacalcyonacea, family Xeniidae), both originating from the Gulf of Aqaba, Red Sea. The main clade of Symbiodiniaceae contained in each coral species was determined in five nubbins from five different mother colonies. For this purpose, nubbins were frozen at −80 °C. DNA was then extracted using the DNeasy Plant mini Kit (Qiagen) for *H. fuscescens* and the PowerSoil DNA Isolation Kit (MO-BIO) for the other species according to Ezzat et al. (2016a,b). *Galaxea fascicularis* mainly hosted *Cladocopium* spp. (clade C; as in Ezzat et al., 2016a,b and Ziegler et al., 2019), while the dominant clade in *H. fuscescens* was *Durusdinium* spp. (clade D; as in Ezzat et al., 2016a,b).

### 2.2 Experimental design

A total of 228 nubbins per species were generated from six parental colonies (38 nubbins per colony) and were distributed in 12 independent 20-L aquaria (3 nubbins per colony and aquarium). Aquaria received a continuous supply of oligotrophic seawater (< 0.5 μM dissolved inorganic nitrogen, DIN, and < 0.1 μM inorganic phosphorus, DIP) at a flow rate of 20 L h^−1^, and a constant temperature of 25 °C ± 0.2 was maintained using submersible resistance heaters (Visi-ThermH Deluxe, Aquarium Systems, France). Submersible pumps ensured proper water mixing in the aquaria. Corals were maintained under an irradiance of 200 ± 10 μmol photons m^−2^ s^−1^ (12:12, light:dark cycle) by 400 W metal halide lamps (HPI-T, Philips, The Netherlands) and were fed once a week with *Artemia salina* nauplii until complete healing.

After the acclimation period, aquaria were divided into two sets of six aquaria each, with two different nutritional treatments being applied: (1) a control treatment (subsequently called “C”), with natural seawater (*ca.* 0.5 μM DIN and 0.1 μM DIP) and (2) a nutrient-enriched treatment (called “NP”), in which the remaining six aquaria were exposed to a combined enrichment of nitrate (NO_3_^−^) and phosphate (PO_4_^3−^), to a final concentration of 2 μM NO_3_^−^ and 0.5 μM PO_4_^3−^. The DIN:DIP ratio was equal to 4–5 in each condition. For the nutrient enriched condition, stock solutions of sodium nitrate (NaNO_3_) and potassium dihydrogen phosphate (KH_2_PO_4_) were continuously pumped from individual batch tanks via a peristaltic pump (Ismatec®) to the experimental tanks. The stock solutions were renewed every 3 days, and flow rate was regulated to 0.3 L h^−1^. Inorganic nutrient concentrations were monitored weekly in triplicates in each tank using an Autoanalyser (Alliance Instrument, AMS, France) according to Aminot and Kérouel (2007).

After 4 weeks under these nutritional conditions (“ctrl” condition), temperature was increased from 25 °C ± 0.2 to 30 °C ± 0.2 over a 10 day-period (+ 0.4 °C d^−1^) and kept constant for 14 days (“heat” stress condition). After 2 weeks under high temperature, all tanks were returned to 25 °C ± 0.2 over a 10 day-period and maintained under these conditions for two additional weeks (“*post-stress*” condition). At the end of each timepoint (*ctrl* at 25 °C, *heat* at 30 °C for 14 days, and *post-stress* after 14 days back at 25 °C) and for both nutrient treatments, *s*ix different sets of measurements were performed (see below), with nubbins sampled from different colonies and different aquaria: (i) a set of five nubbins for the measurements of oxygen fluxes, symbiont density and total chlorophyll content; (ii) an additional set of four nubbins for oxygen fluxes of freshly isolated symbionts (FIZ); (iii) a set of 15 nubbins for the inorganic nutrient uptake rates (3 nutrients tested independently, *i.e n* = 5); (iv) a set of five nubbins for the energy reserves (protein, lipid and carbohydrate contents); (v) a set of four nubbins for isotopic measurements; (vi) a last set of five nubbins for CNP tissue content of the holobiont.

### 2.3 Photosynthesis and respiration rates of holobiont and freshly isolated symbionts (FIZ)

At the end of each timepoint (*ctrl*, *heat* and *post-stress*), net photosynthesis (P_n_) and respiration (R) rates were determined on five nubbins per species and nutrient treatment. Each nubbin was placed in a 50-mL hermetically-sealed glass chamber filled with 0.45-μm filtered seawater (FSW), maintained under the appropriate temperature (25 °C or 30 °C) in a thermostatic bath and was continuously stirred with a magnetic bar. Measurements were performed both at 200 μmol photons m^−2^ s^−1^ and in the dark. The chambers were equipped with a Unisense optode (oxygen-sensitive minisensor) connected to the Oxy-4 software (Chanel fiber-optic oxygen meter, Presens, Regensburg, Germany). Optodes were calibrated at 0 and 100% O_2_ with nitrogen-saturated and air-saturated seawater, respectively. P_n_ and R were determined by regressing O_2_ production or consumption against time, and gross photosynthesis (P_g_) was assessed by adding the absolute value of R to P_n_. The daily percentage contribution of photosynthesis to the holobiont’s respiratory needs (referred to as P:R ratio in the following sections) was computed as P_C_/R_C_, where P_C_ is the total daily grams of carbon per gram of ash-free dry weight (AFDW), acquired over 12 h of gross photosynthesis (P_g_ × 12 / PQ), and R_C_ represents the daily grams of carbon respired per gram of AFDW (R × 24 × RQ). The photosynthetic and respiratory quotients (PQ and RQ) are, respectively, equal to 1.1 mol O_2_/mol C and 0.8 mol C/mol O_2_ (Muscatine et al., 1981). At the end of the measurements, nubbins were frozen at −20 °C for the later determination of total chlorophyll and symbiont density.

The respiration rates of FIZ (R_S_) were also measured for four nubbins per timepoint, species and nutrient treatment, using the same technique as for the coral holobionts as described in Tremblay et al. (2012). The host respiration (R_H_) was estimated by subtracting R_S_ from R_C_.

### 2.4 H^13^CO_3_ labelling experiments

We assessed the acquisition of carbon and translocation of photosynthates within the coral-dinoflagellate symbiosis at the end of each timepoint (*ctrl*, *heat* and *post-stress*) using the H^13^CO_3_ labelling technique according to Tremblay et al. (2012). For each nutrient treatment and species, four nubbins from different colonies were placed in individual beakers filled with 200 mL FSW, enriched with NaH^13^CO_3_ (98 atom %^13^C, no. 372382, Sigma-Aldrich, St Louis, MO, USA) at a final concentration of 0.6 mM. Additionally, two control nubbins were incubated in non ^13^C-enriched seawater for natural isotopic abundance. After a 5-h incubation (“pulse”), nubbins were transferred to beakers containing non ^13^C-enriched FSW for a “chase” period of 19 h and subsequently frozen at −20 °C. Coral nubbins were then processed as detailed in section 6. *Tissue parameters and sample processing*, and the host and symbiont fractions were separated by centrifugation and freeze-dried. The %^13^C enrichment in the host and the symbionts fractions was then quantified using a EA-IRMS (Sercon, UK). Detailed calculations are described in Tremblay et al. (2012) and available as Supplementary material.

### 2.5 Dissolved inorganic nutrients uptake rates

Uptake rates of three inorganic nutrients, namely ammonium (NH_4_^+^), nitrate (NO_3_^−^) and phosphate (PO_4_^3−^), were assessed at the end of each time point (*ctrl*, *heat* and *post-stress*) (Godinot et al., 2011). For each nutrient tested, five nubbins per species and nutrient treatment were incubated in individual 200-mL beakers with 0.45-μm FSW continuously stirred and maintained at the corresponding experimental temperature (25 °C or 30 °C). Three control beakers per treatment, without coral nubbins, were used to account for potential change in water chemistry due to adsorption onto beaker surface, consumption by microbial activity, or air contamination. After a 20-min acclimation period, seawater was enriched with a stock solution of either ammonium chloride (NH_4_Cl), sodium nitrate (NaNO_3_), or potassium dihydrogen phosphate (KH_2_PO_4_), in order to reach a final concentration of 2 μM N or 1 μM P. Immediately after the enrichment, and then subsequently after 15, 30, 45, 60 and 90 min, subsamples of 10 mL of water (Aminot and Kérouel, 2007). Uptake rates of each inorganic nutrient were calculated as the difference in concentration between the 0- and 90-min sampling timepoints, after data correction for the diminution of the beakers volume and verification of the linearity in the depletion during the experiment. All data were normalized to AFDW.

### 2.6 Tissue parameters and sample processing

All data were normalized to AFDW (Pupier et al., 2018), since skeletal surface area was impossible to retrieve from the soft coral *H. fuscescens*. Samples were processed as described in Pupier et al. (2018). Briefly, coral nubbins were freeze-dried then crushed into powder and homogenized with Milli-Q water. For *G. fascicularis*, tissue was separated from the skeleton using a water-pick before being freeze-dried.

#### 2.6.1 Symbiont density and total chlorophyll content

For all nubbins, a 100-μL subsample of the tissue slurry was collected for the measurement of dinoflagellate density in triplicate, using a Z1 Coulter Particle Counter (Beckman Coulter, USA). For the nubbins used in oxygen fluxes measurements, a second subsample of 5 mL was used for total chlorophyll a and c_2_ concentration determination. After a centrifugation at 8,000 *g* and 4 °C for 10 min, supernatant was removed, and symbionts were resuspended in 5 mL 100% acetone and placed in the dark at 4 °C overnight. Absorbances at 616 nm and 663 nm were measured using a Xenius spectrophotometer (SAFAS®, Monaco) and the total chlorophyll concentration was determined according to Jeffrey and Humphrey (1975).

#### 2.6.2 Tissue energy reserves

Five nubbins per species and nutrient treatment were freeze-dried at the end of each timepoint for quantification of coral tissue energy reserves (namely protein, lipid, and carbohydrate contents) by colorimetric assays using a spectrophotometer (Xenius, SAFAS®, Monaco). Coral tissue was extracted as previously described, and a 500 μL subsample of the tissue slurry was collected for protein quantification, while the remaining homogenate was freeze-dried for lipid and carbohydrate analyses. Protein concentration was measured by incubating the 500-μL subsample in 1 mol L^−1^ NaOH at 90 °C for 30 min, followed by quantification with the BC assay kit (Smith et al., 1985) using bovine serum albumin standards (0–2,000 μg mL^−1^). Absorbance was recorded at 562 nm after a 30-min incubation at 60 °C in microplates. Lipid content was determined following a modified method from Cheng et al. (2011). Ten mg of dry tissue were extracted in chloroform:methanol (2:1, v/v), centrifuged, and the supernatant transferred to quartz microplates. Cholesterol was used for standards (0–200 μg). After solvent evaporation at 90 °C, sulfuric acid was added and incubated for 20 min before measuring the absorbance at 540 nm. Vanillin–phosphoric acid reagent was then added, and absorbance was measured again at 540 nm, correcting for background coloration. Carbohydrates were quantified following Dubois et al. (1956). Five mg of dried tissue were incubated with 5% phenol, treated with concentrated sulfuric acid, centrifuged, and compared to a D-glucose calibration curve (0–125 mg L^−1^). Absorbance was measured at 490 nm in a quartz microplate.

Total tissue energy (kJ g^−1^ AFDW) was calculated by converting protein, lipid, and carbohydrate contents into joules using their respective enthalpies of combustion (−17.5, −39.5, and −23.9 J mg^−1^; Gnaiger and Bitterlich, 1984).

#### 2.6.3 C, N, and P content

Five nubbins per species and nutrient treatment were freeze-dried at the end of each timepoint for later determination of carbon (C), nitrogen (N) and phosphorus (P) content. For C and N analyses, subsamples of freeze-dried tissue (600 μg) were placed in a tin cap and analyzed with an elemental analyzer coupled to an isotope ratio mass spectrometer (EA-IRMS Integra2, Sercon, UK). For the quantification of the P content, 5 mg of freeze-dried tissue were placed in Pyrex tubes and combusted at 500 °C for 2 h to oxidize organic matter. After cooling to room temperature, the resulting ash was dissolved in 5 mL of 0.2 M HCl and heated at 85 °C for 45 min to ensure complete solubilization of inorganic phosphate. Samples were then allowed to cool, diluted with 5 mL of Milli-Q water, and analyzed using a continuous-flow autoanalyzer (AA3, SEAL Analytical) according to Aminot and Kérouel (2007). Prior to analysis, samples were diluted tenfold with Milli-Q water. Phosphate concentrations were quantified using a standard curve with NaH₂PO₄ (0.5, 1, 3, and 5 μmol L^−1^).

### 2.7 Statistical analysis

All statistical analyses were performed in RStudio (version 2025.05.1 + 513). Data are expressed as mean ± standard deviation. Outliers were identified using Grubb’s test and removed when significant (*p* < 0.05). The assumptions of normality and homoscedasticity were assessed with the Shapiro–Wilk and Levene’s tests, supplemented by graphical inspection of residuals. When assumptions were met, a three-way ANOVA was used to test the effect of nutrient treatment, temperature and species on physiological parameters, followed by Tukey’s HSD *post hoc* comparisons when significant differences were detected. If assumptions were not met, a Kruskal–Wallis test followed by Dunn’s test were performed, independently testing the effects of nutrient treatment, temperature and species. For all analyses, differences were considered statistically significant when *p* < 0.05, and details are available as Supplementary Tables S1–S3. Data normalized to symbiont density (for oxygen fluxes, carbon incorporation in symbionts and translocation to host, and inorganic nutrient uptake) are available as Supplementary Figure S4.

## 3 Results

### 3.1 Physiological parameters

#### 3.1.1 Effect of nutrient enrichment alone

**Figure 1:**
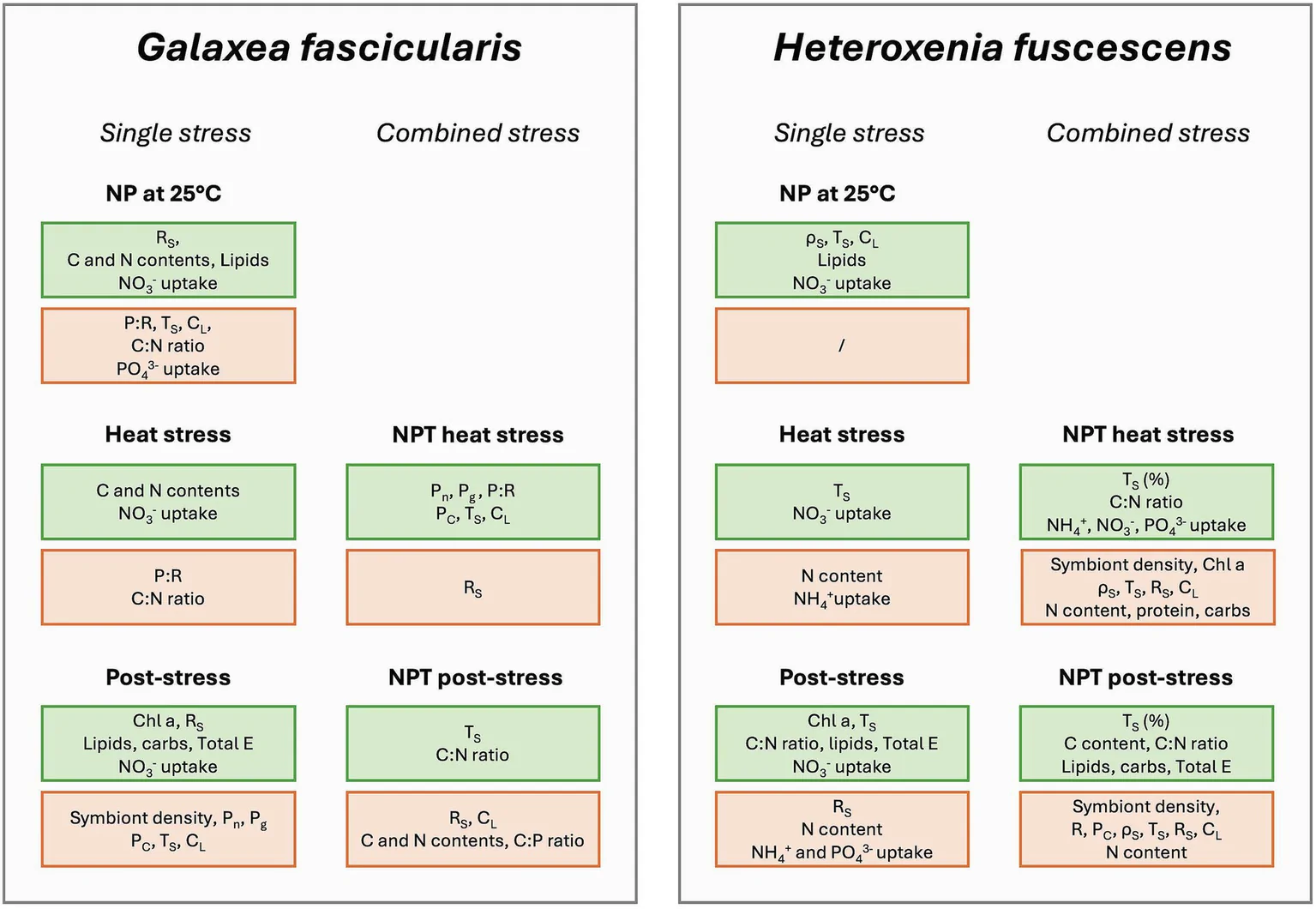
Summary of the main physiological responses and inorganic nutrient uptake across experimental treatments. Symbols are defined in the main text. Green and red boxes represent a significant increase and decrease in the physiological parameters, respectively.

A summary of the main outcomes of nutrient enrichment on coral physiology is illustrated in Figure 1 and described in detail below.

**Figure 2:**
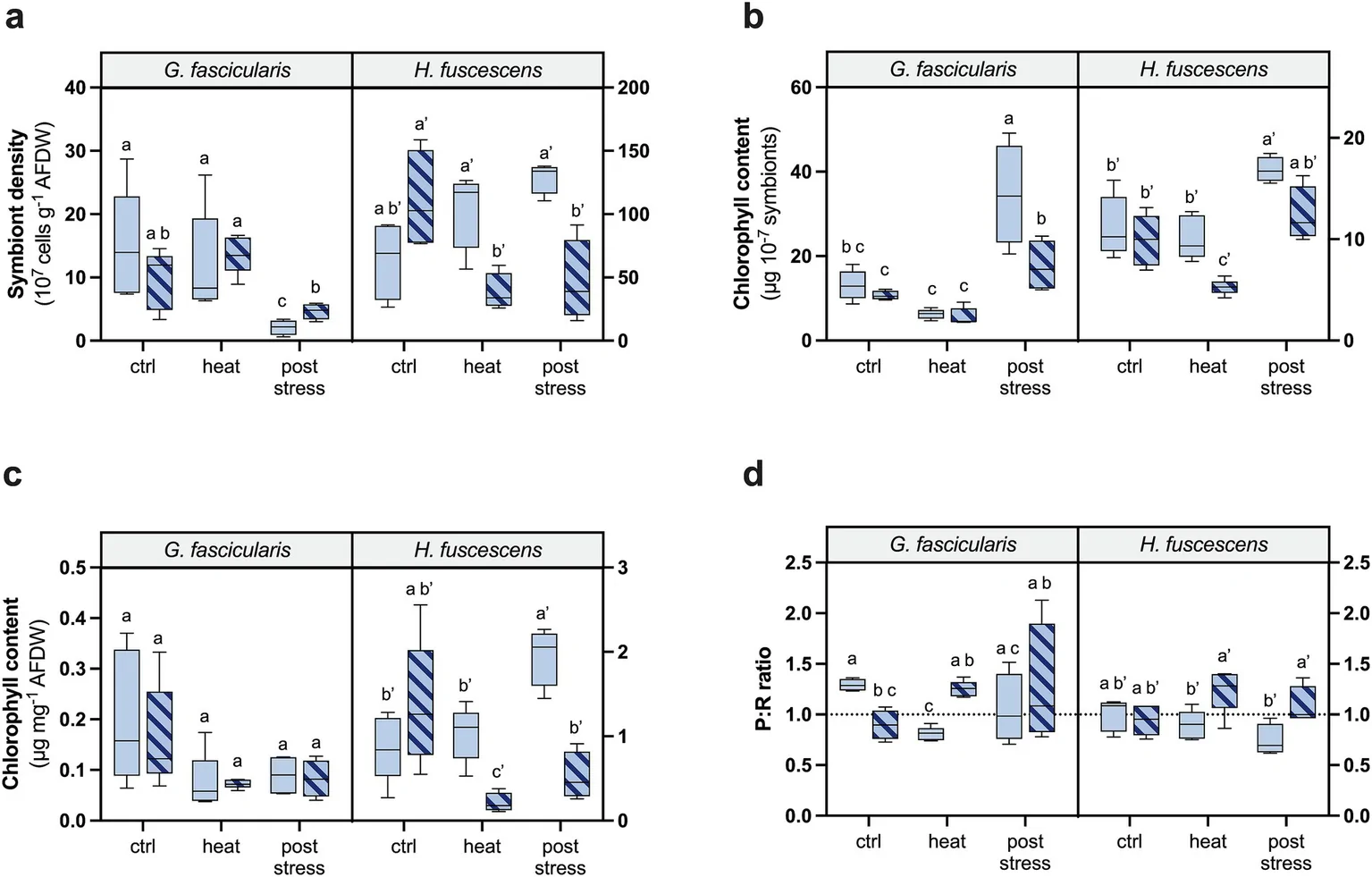
Effect of nutrient enrichment and warming on coral physiological parameters. Boxplots show **(a)** symbiont density (×10^7^ cells g^−1^ AFDW), total chlorophyll a + c_2_ content normalized **(b)** to symbiont cells (μg 10^−7^ cells) and **(c)** to AFDW (μg mg^−1^ AFDW) and **(d)** the P:R ratio for *Galaxea fascicularis* and *Heteroxenia fuscescens* under different experimental conditions. Plain blue boxplots represent corals not enriched in nutrients (“C” corals) while stripped blue boxplots represent corals exposed to nutrient enrichment (“NP corals”). “C*trl*” refers to control conditions of temperature at 25 °C, “heat” refers to an exposure to 30 °C for 14 days, and “post-stress” refers to the recovery phase, with temperature going back to 25 °C for 14 days. Data shown as mean ± SD (*n* = 5 nubbins per species and condition). Letters indicate significant differences between treatments, for each species independently.

**Figure 3:**
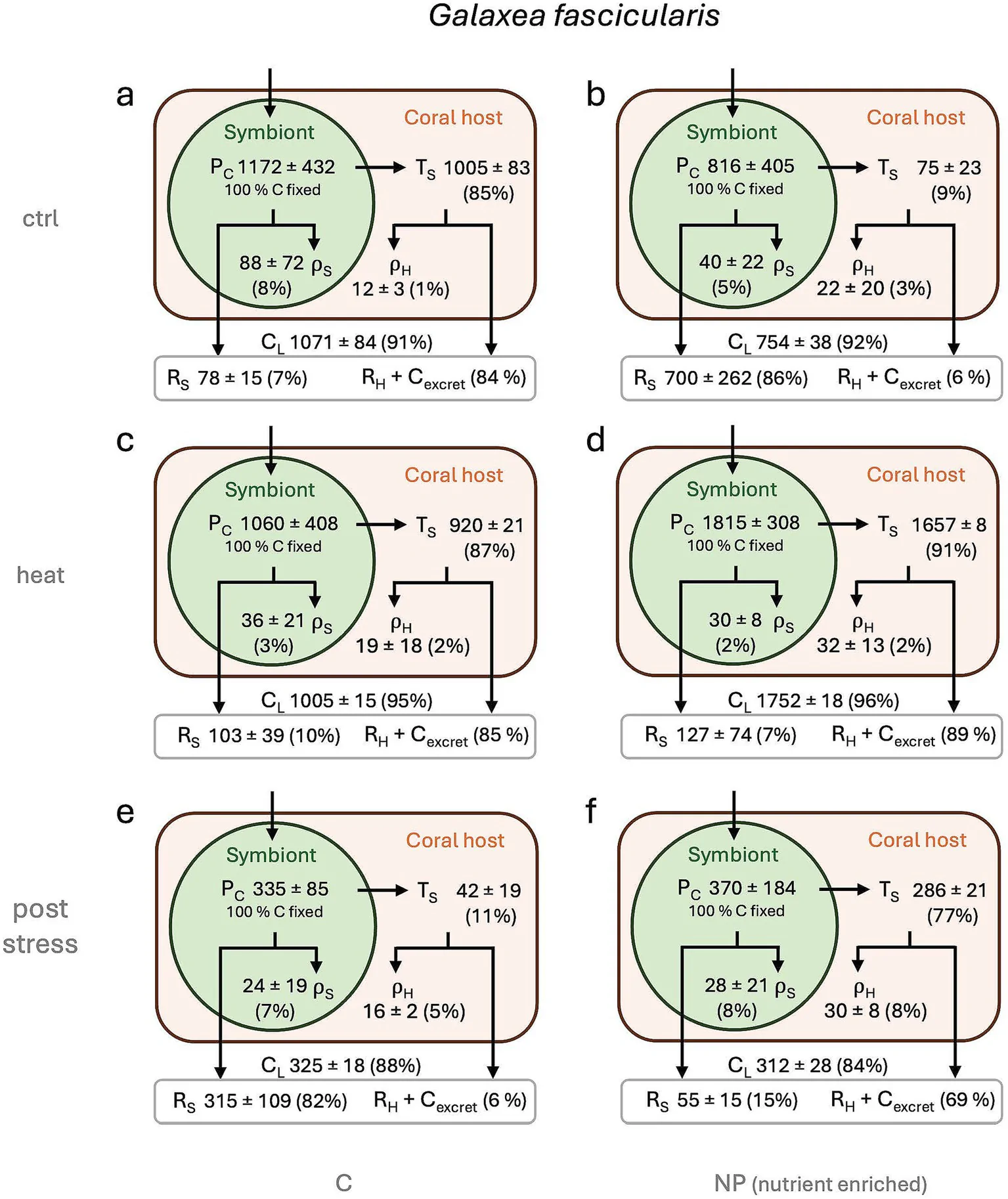
Mass-balanced carbon budgets for the hard coral *Galaxea fascicularis* based on ^13^C tracer experiments after 24 h. Symbols are defined in the main text. Rates are expressed as μg C g^−1^ AFDW h^−1^ and reported as mean ± SD; *n* = 4 for ^13^C-based rates (*ρS*, *ρH*, and *TS*) and *n* = 5 for photosynthesis (*PC*) and respiration (*RS* and *R*). *RH* values are shown without SD because they were calculated from mean values of the other terms. Carbon budget at 25 °C are shown in **(a,b)**, at 30 °C in **(c,d)**, and in post stress phase back at 25 °C in **(e,f)**; budgets represented in **(a,c,e)** are in controlled nutrient conditions, contrary to **(b,d,f)** exposed to nutrient enrichment. Panel layout: **(a,b)** Control at 25 °C; **(c,d)** Heat stress at 30 °C; **(e,f)** Post-stress phase back at 25 °C. For each temperature, control nutrient conditions are shown in the left panel **(a,c,e)** and nutrient-enriched conditions in the right panel **(b,d,f)**.

**Figure 4:**
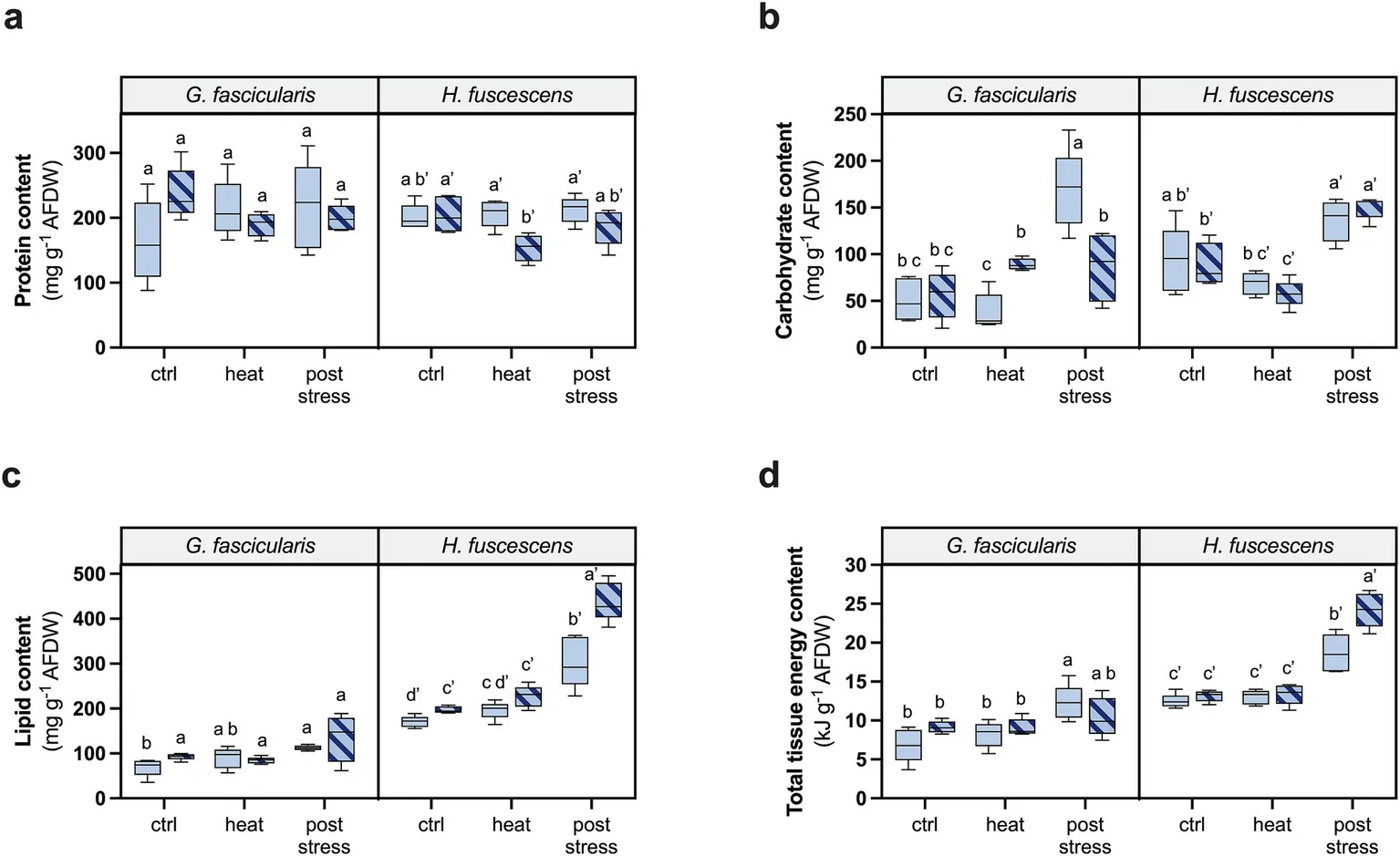
Effect of nutrient enrichment and warming on coral energy reserves. Boxplots show **(a)** total protein, **(b)** total carbohydrate, and **(c)** total lipid content (mg g^−1^ AFDW), and **(d)** total tissue energy content (kJ g^−1^ AFDW) for *Galaxea fascicularis* and *Heteroxenia fuscescens* under different experimental conditions. Plain blue boxplots represent corals not enriched in nutrients (“C” corals) while stripped blue boxplots represent corals exposed nutrient enrichments (“NP corals”). “*ctrl*” refers to control conditions of temperature at 25 °C, “heat” refers to an exposure to 30 °C for 14 days, and “post-stress” refers to the recovery phase, with temperature going back to 25 °C for 14 days. Data shown as mean ± SD (*n* = 5 nubbins per species and condition). Letters indicate significant differences between treatments, for each species independently.

In *Galaxea fascicularis*, nutrient enrichment did not significantly affect symbiont density (Figure 2a), chlorophyll content (Figures 2b,c), and oxygen fluxes (net and gross photosynthesis, holobiont respiration; P_n_, P_g_ and R; Supplementary Figures S1a–c) (*p* > 0.05). Therefore, in agreement with the oxygen fluxes, total carbon fixation measured using ^13^C-bicarbonate (P_C_; μg C g^−1^ AFDW h^−1^) remained comparable to the control condition (*p* > 0.05, Figures 3a,b; Supplementary Figure S2a). Regarding the carbon budget (Figures 3a,b; Supplementary Figure S2), carbon incorporation rates into symbionts (ρ_S_) and host (ρ_H_) remained also comparable to the control condition (*p* > 0.05). However, carbon translocation from symbionts to host (T_S_) dropped by 90% (*p* < 0.0001) representing only 9% of the total fixed carbon in nutrient-enriched nubbins (“NP” nubbins, *vs.* 85% in control “C” nubbins; *p* < 0.0001). This was associated with a 9-fold increase in symbiont respiration (R_S_, from 7% to 86% of total fixed carbon; *p* < 0.05), in agreement with the significant decrease in P:R ratio compared to controls (−30%; *p* < 0.01; Figure 2d), which fell below 1. There was also a 30% decrease in carbon loss (C_L_; *p* < 0.001), although relative loss remained stable (92% of total fixed carbon). Thus, under nutrient enrichment, carbon loss occurred predominantly via symbiont respiration rather than host respiration and excretion (observed for C nubbins). Tissue carbon and nitrogen contents increased by 25% and 40%, respectively (both *p* < 0.05; Supplementary Figures S3a,b), resulting in a lower tissue C:N ratio compared to C nubbins (−11%; *p* < 0.05; Supplementary Figure S3d). However, tissue phosphorus content and C:P and N:P ratios (Supplementary Figures S3c,e,f) remained comparable to control conditions (*p* > 0.05). While protein and carbohydrate contents were not affected by nutrient enrichment (*p* > 0.05; Figures 4a,b), lipid reserves increased by 34% (*p* < 0.05; Figure 4c), without significantly affecting total tissue energy content (*p* > 0.05; Figure 4d).

**Figure 5:**
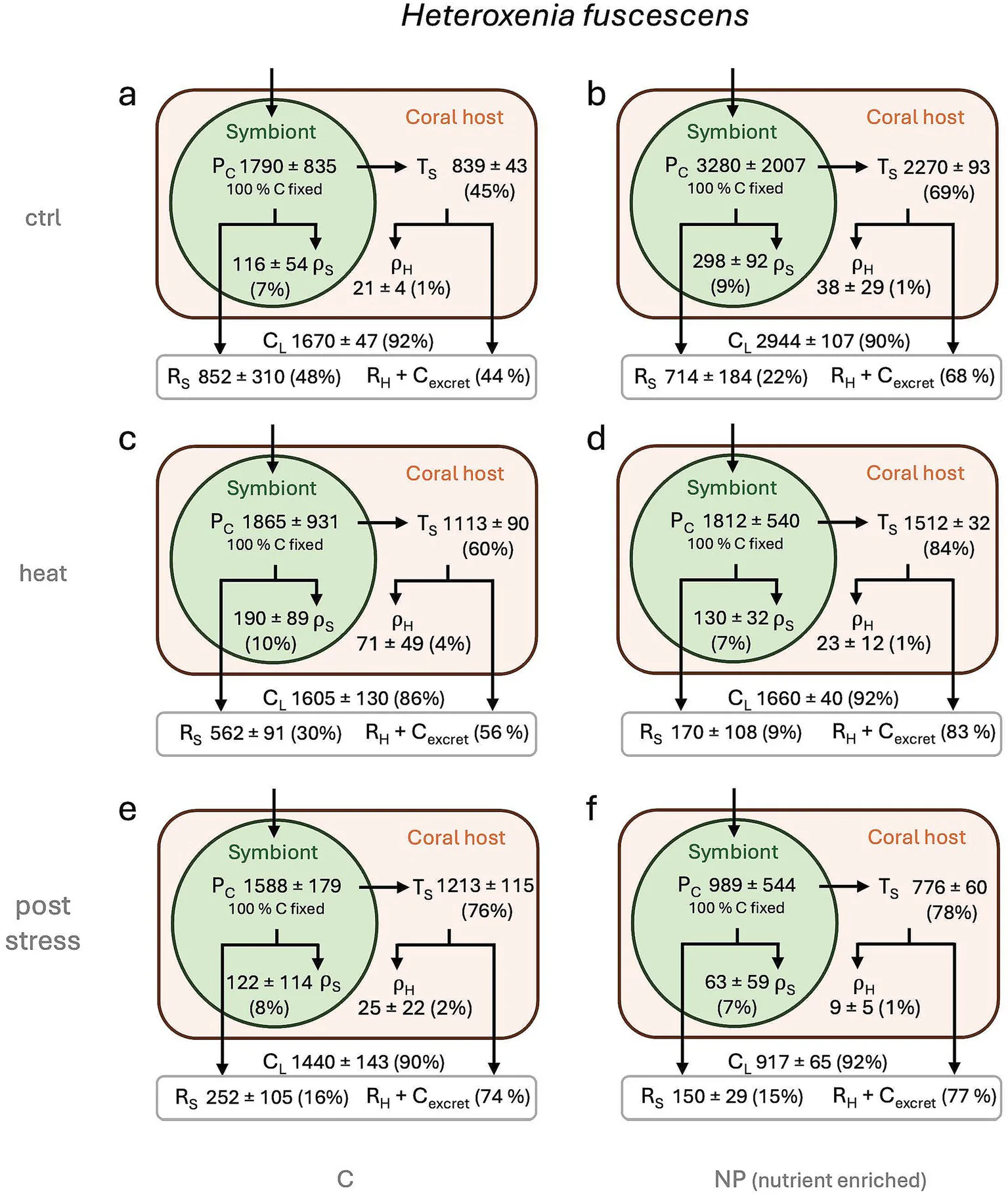
Mass-balanced carbon budgets for the soft coral *Heteroxenia fuscescens* based on ^13^C tracer experiments after 24 h. Symbols are defined in the main text. Rates are expressed as μg C g^−1^ AFDW h^−1^ and reported as mean ± SD; *n* = 4 for ^13^C-based rates (ρ_S_, ρ_H_, and T_S_) and *n* = 5 for photosynthesis (P_C_) and respiration (R_S_ and R). R_H_ values are shown without SD because they were calculated from mean values of the other terms. Carbon budget at 25 °C are shown in **(a,b)**, at 30 °C in **(c,d)**, and in post stress phase back at 25 °C in **(e,f)**; budgets represented in **(a,c,e)** are in controlled nutrient conditions, contrary to **(b,d,f)** exposed to nutrient enrichment. Panel layout: **(a,b)** Control at 25 °C; **(c,d)** Heat stress at 30 °C; **(e,f)** Post-stress phase back at 25 °C. For each temperature, control nutrient conditions are shown in the left panel **(a,c,e)** and nutrient-enriched conditions in the right panel **(b,d,f)**.

In *H. fuscescens,* nutrient enrichment did not significantly affect symbiont density (Figure 2a), chlorophyll content (Figures 2b,c), P:R ratio (Figure 2d) and oxygen fluxes (net and gross photosynthesis, holobiont respiration; P_n_, P_g_ and R; Supplementary Figures S1a–c) (*p* > 0.05). Therefore, in agreement with the oxygen fluxes, total carbon fixation measured using ^13^C-bicarbonate (P_C_; μg C g^−1^ AFDW h^−1^) remained comparable to the control condition (*p* > 0.05; Figures 5a,b; Supplementary Figure S2a). Regarding the carbon budget (Figures 5a,b; Supplementary Figure S2), nutrient enrichment led to a 2.5-fold increase in ρ_S_ (*p* < 0.05) and T_S_ (*p* < 0.0001), without affecting R_S_. The fraction of fixed carbon translocated increased from 45 to 69% (*p* < 0.001). This increased carbon translocation did not significantly affect ρ_H_ (*p* > 0.05) and thus led to a 75% increase in C_L_ (*p* < 0.0001), though the relative proportion of carbon lost remained stable (90% of fixed carbon). Nutrient enrichment did not affect tissue carbon, nitrogen and phosphorus contents, nor C:N, C:P and N:P ratios (*p* > 0.05; Supplementary Figure S3). However, lipid content increased by 15% in enriched nubbins compared to controls (*p* < 0.01; Figure 4c), while protein and carbohydrate contents remained unchanged (*p* > 0.05; Figures 4a,b). Consequently, total tissue energy content did not differ between treatments (*p* > 0.05; Figure 4d).

#### 3.1.2 Effect of heat stress

In *G. fascicularis*, short-term exposure to 30 °C for 14 days did not significantly affect symbiont density (Figure 2a), chlorophyll content (Figures 2b,c), oxygen fluxes (Supplementary Figures S1a–c), any parameter of the carbon budget: P_C_, ρ_S_, ρ_H_, T_S_, and C_L_ (Figures 3a,c), or energy reserves (Figure 4). However, the P:R ratio significantly decreased by 37% (*p* < 0.05; Figure 2d). While phosphorus tissue content (and associated C:P and N:P ratios in tissue; Supplementary Figures S3c,e,f) did not change (*p* > 0.05), there was a 37 and 64% increase in the carbon and nitrogen tissue contents, respectively (*p* < 0.01; Supplementary Figures S3a,b), leading to a lower C:N tissue ratio at 30 °C (−17%; *p* < 0.05; Supplementary Figure S3d).

During the post-stress phase (14 days back at 25 °C), *G. fascicularis* exhibited significant bleaching, with an 86% decrease in symbiont density (*p* < 0.05; Figure 2a) despite stable chlorophyll content per AFDW (*p* > 0.05; Figure 2c), inducing a 160% increase in chlorophyll per symbiont cell (*p* < 0.001; Figure 2b). This was accompanied by a 3-fold decline in photosynthetic rates (P_n_, P_g_; *p* < 0.05; Supplementary Figures S1a,b), indicating incomplete recovery despite the P:R ratio returning to 1 (Figure 2d). These changes agree with the carbon budget (Figures 3a,c,e; Supplementary Figure S2), which showed a significant decrease in P_C_ while R_S_ increased by 3-fold (both *p* < 0.05), causing a sharp drop in T_S_ (20-fold decrease; *p* < 0.0001). Indeed, the fraction of carbon translocated dropped from 85 to 11% (*p* < 0.0001), while R_S_ rose to 82% of total fixed carbon (vs. 7–10% in control and heat conditions). Host incorporation (ρ_H_) remained stable across temperature conditions, and C_L_ decreased by 70% (*p* < 0.0001), inducing a decrease in relative carbon loss (88% of total fixed carbon *vs.* 95% at 30 °C; *p* < 0.05). Thus, as for nutrient enrichment alone, the post-stress phase was overall characterized by minimal carbon transfer to the host and predominant symbiont respiration, suggesting persistent disruption in carbon allocation and energy exchange between symbionts and host after a heat stress. *Galaxea fascicularis* also exhibited marked changes in tissue composition and energy storage. Tissue carbon and nitrogen contents decreased by 29% and 47%, respectively, compared to the heat-stress phase (*p* < 0.01 and *p* < 0.001; Supplementary Figures S3a,b), resulting in a higher C:N ratio (+36%; *p* < 0.001; Supplementary Figure S3d). Thus, tissue elemental composition returned to control levels (*p* > 0.05). Protein content remained stable across temperature treatments (*p* > 0.05; Figure 4a), but lipid and carbohydrate contents significantly increased during the post-stress phase (+63% and +230% relative to control levels, respectively; *p* < 0.01 and *p* < 0.001; Figures 4b,c), leading to an 80% increase in total tissue energy content compared to control conditions (*p* < 0.01; Figure 4d).

In *H. fuscescens*, short-term exposure to 30 °C did not significantly affect symbiont density, chlorophyll content, oxygen fluxes, or any parameter of the carbon budget: P_C_, ρ_S_, ρ_H_, and C_L_, (Figures 5a,c), except T_S_, the amount of carbon translocated, which increased by 25% (from 45% to 60% of total fixed carbon; *p* < 0.05; Figures 5a,c; Supplementary Figure S2). Contrary to *G. fascicularis*, *H. fuscescens* did no bleach during the post-stress phase. Instead, chlorophyll content significantly increased compared to controls (+126% per AFDW, *p* < 0.01; +50% per symbiont, *p* < 0.05; Figures 2b,c). The translocation rate increased even further (*p* < 0.01; Figure 5e; Supplementary Figure S2), reaching 76% of fixed carbon (*p* < 0.001), while R_S_ dropped by 70% (relative fraction represented 16% of fixed carbon vs. 48% in control; *p* < 0.01; Supplementary Figure S2) (Figures 5a,c,e). Nitrogen content in tissue declined (−21% *vs.* control; *p* < 0.0001; Supplementary Figure S3b), resulting in a 30% increase in the C:N ratio (*p* < 0.0001; Supplementary Figure S3d). Energy reserves increased, with lipid content rising by 78% compared to control levels (*p* < 0.001; Figure 4c) and carbohydrate content by 46% (although only significant relative to the heat-stress phase; Figure 4b). Consequently, total tissue energy content was 49% higher than in C nubbins (*p* < 0.001; Figure 4d).

A summary of the main outcomes of thermal stress on coral physiology is illustrated in Figure 1.

#### 3.1.3 Combined effect of nutrient enrichment and temperature

A summary of the main outcomes of combined nutrient enrichment and thermal stress on coral physiology is illustrated in Figure 1 and described in detail below.

In *G. fascicularis*, high temperature and nutrient enrichment doubled photosynthetic rates (P_n_: *p* < 0.05; P_g_: *p* < 0.01; Supplementary Figures S1a,b). Consequently, at 30 °C, nutrient-enriched and thermally stressed corals (“NPT” nubbins) exhibited significantly higher photosynthesis than control nubbins (+230% for P_n_, +71% for P_g_; *p* < 0.05), resulting in a 55% higher P:R ratio (*p* < 0.0001; Figure 2d). These results are in agreement with the carbon budget using ^13^C-bicarbonate (Figures 3b,d; Supplementary Figure S2), where P_C_ doubled (*p* < 0.01), and corresponded to a 20-fold increase in T_S_ (*p* < 0.0001), reaching the highest rate across nutrient and temperature condition for this species (1,815 μg C g^−1^ AFDW h^−1^), and resulting in a 10-fold increase in the proportion of fixed carbon translocated (from 9% to 91% at 30 °C; *p* < 0.0001). The greater amount of carbon available for translocation was linked to a 5-fold reduction in R_S_ (*p* < 0.01), leading to a twofold increase in C_L_ (*p* < 0.0001). As a result, at 30 °C NPT nubbins had 70%–80% higher P_C_, T_S_ and C_L_ than C nubbins (*p* < 0.05, *p* < 0.0001, and *p* < 0.0001, respectively). In addition, coral nubbins showed a 25% lower nitrogen tissue content (*p* < 0.05; Supplementary Figure S3b), 20% higher C:N ratio (*p* < 0.01; Supplementary Figure S3d) and two times higher phosphorus content (+123%, *p* < 0.001; Supplementary Figure S3c) than C nubbins.

During the post-stress phase, NPT nubbins showed delayed but moderate bleaching (−67% symbiont density; *p* < 0.05; Figure 2a) but increased chlorophyll content per symbiont (*p* < 0.01; Figure 2b). As the bleaching was less severe than under heat stress alone, nubbins contained twice as many symbionts but two-fold lower chlorophyll content per cell than C nubbins (*p* < 0.05; Figures 2a,b). Photosynthetic rates (P_n_ and P_g_) dropped by 80–85% (*p* < 0.001; Supplementary Figures S1a,b), and respiration by 74% (*p* < 0.05, Supplementary Figure S1c), returning to control levels. This is in agreement with the parameters measured for the carbon budget (Figures 3b,d,f; Supplementary Figure S2), where P_C_ returned to control levels (−80% compared to heat stress; *p* < 0.001), resulting in a decrease in T_S_ (*p* < 0.0001) though still four times higher than controls (*p* < 0.0001). Due to the lower P_C_, C_L_ per AFDW decreased by 60%–80% (*p* < 0.0001), while R_S_ stayed lower than controls (*p* < 0.001). Consequently, in post stress phase, nubbins had higher T_S_ and ρ_H_, and lower R_S_ compared to C nubbins (all *p* < 0.05). Carbon and nitrogen tissue contents were reduced by 20 and 40%, respectively, compared to control levels (*p* < 0.05 and *p* < 0.001; Supplementary Figures S3a,b), resulting in a higher C:N ratio (+40%; *p* < 0.001; Supplementary Figure S3d) and a lower C:P ratio in tissue (−32%; *p* < 0.05; Supplementary Figure S3e). No significant effect of combined nutrient enrichment and warming on phosphorus tissue content (Supplementary Figure S3c), N:P ratio (Supplementary Figure S3f) or energy reserves (protein, lipid and carbohydrate contents, and total tissue energy content; Figures 4a–d) were observed.

In contrast, *H. fuscescens* underwent immediate and severe bleaching under combined stress (−70% symbiont density; −50% to 85% chlorophyll per AFDW or per cell; all *p* < 0.05; Figures 2a–c). Thus, nubbins displayed significantly fewer symbionts and lower chlorophyll than C nubbins (*p* < 0.05 and *p* < 0.01). Despite this bleaching, oxygen fluxes and P:R ratio remained statistically comparable to controls (*p* < 0.05; Figure 2d, Supplementary Figure S1). In agreement with these results, P_C_ (Figures 5b,d; Supplementary Figure S2) did not significantly change compared to control condition, although showing some decrease inducing a 2-fold decrease in ρ_S_ (*p* < 0.05) at 30 °C. There was a 4-fold decrease in R_S_ (*p* < 0.01; only 9% of carbon fixed) and lower T_S_ per AFDW (*p* < 0.0001). Despite less photosynthates translocated, the relative fraction of total fixed carbon translocated increased from 69 to 84% (*p* < 0.01). Carbon loss per AFDW also decreased by 44% (*p* < 0.0001), while ρ_H_ remained stable. In addition, combined stress at 30 °C resulted in lower nitrogen tissue content (−26%; *p* < 0.01; Supplementary Figure S3b) and a higher C:N ratio (+30%; *p* < 0.001; Supplementary Figure S3d), accompanied by reductions in protein and carbohydrate contents (−25% to 35%; both *p* < 0.05; Figures 4a,b). Thus, at 30 °C NPT nubbins displayed lower nitrogen and protein content in tissue, as well as higher C:N ratio compared to C nubbins (−21%, *p* < 0.01, Supplementary Figure S3b; −26%, *p* < 0.01, Figure 4a; +23%, *p* < 0.001, Supplementary Figure S3d; respectively).

During the post-stress phase, NPT nubbins failed to recover their symbiont density (−60%; *p* < 0.05), but chlorophyll increased by 60% (*p* < 0.01), returning to control levels (Figures 2a–c). Oxygen fluxes showed delayed stress effects, as P_g_ and R dropped by 70% (*p* < 0.05; Supplementary Figures S1b,c). As a result, NPT nubbins showed lower symbiont density (−65%, *p* < 0.05), chlorophyll per AFDW (−73%, *p* < 0.001), P_g_ (−38%, *p* < 0.05), and R (−58%, *p* < 0.01) than C nubbins, while maintaining a 45% higher P:R ratio (*p* < 0.05). In agreement with oxygen fluxes, P_C_ further decreased (3-fold vs. control, *p* < 0.05), resulting in lower ρ_S_, T_S_, R_S_ and C_L_ compared to controls (all *p* < 0.01) (Figures 5b,d,f; Supplementary Figure S2). Nevertheless, the proportion of carbon translocated remained higher (78% of fixed carbon vs. 69% in control; *p* < 0.05). As a result, NPT nubbins in post stress phase had lower T_S_ and C_L_ than C nubbins (both *p* < 0.001), due to their lower P_C_ (*p* < 0.05). Across treatments, ρ_S_ accounted for 7%–10% of P_C_, and relative C_L_ remained stable (86%–92% of P_C_), suggesting that *H. fuscescens* maintained an effective carbon allocation strategy even under combined stress and despite reduced productivity. Carbon tissue content increased by 9% compared to control (*p* < 0.05; Supplementary Figure S3a), inducing a 48% increase in C:N tissue ratio (*p* < 0.0001; Supplementary Figure S3d), as nitrogen content in tissue remained lower than control levels (−24%; *p* < 0.001; Supplementary Figure S3b). No effect of combined stress on C:P and N:P tissue ratios of *H. fuscescens* were observed, nor on phosphorus content in tissue (*p* > 0.05; Supplementary Figures S3c,e,f). Energy reserves appeared to be boosted in the post-stress phase, with protein content returning to control levels (*p* > 0.05; Figure 4a), while carbohydrate and lipid contents increased by 70 and 125%, respectively (*p* < 0.001 and *p* < 0.0001; Figures 4b,c). This resulted in a near doubling of total tissue energy content compared to control levels (*p* < 0.0001; Figure 4d). Therefore, compared to C nubbins, post-stress NPT nubbins displayed higher carbon content (+9%, *p* < 0.01), C:N ratio (+18%, *p* < 0.01), lipid content (+ 44%; *p* < 0.01) and total tissue energy reserves (+30%; *p* < 0.01).

### 3.2 Uptake of inorganic nutrients

#### 3.2.1 Effect of nutrient enrichment alone

**Figure 6:**
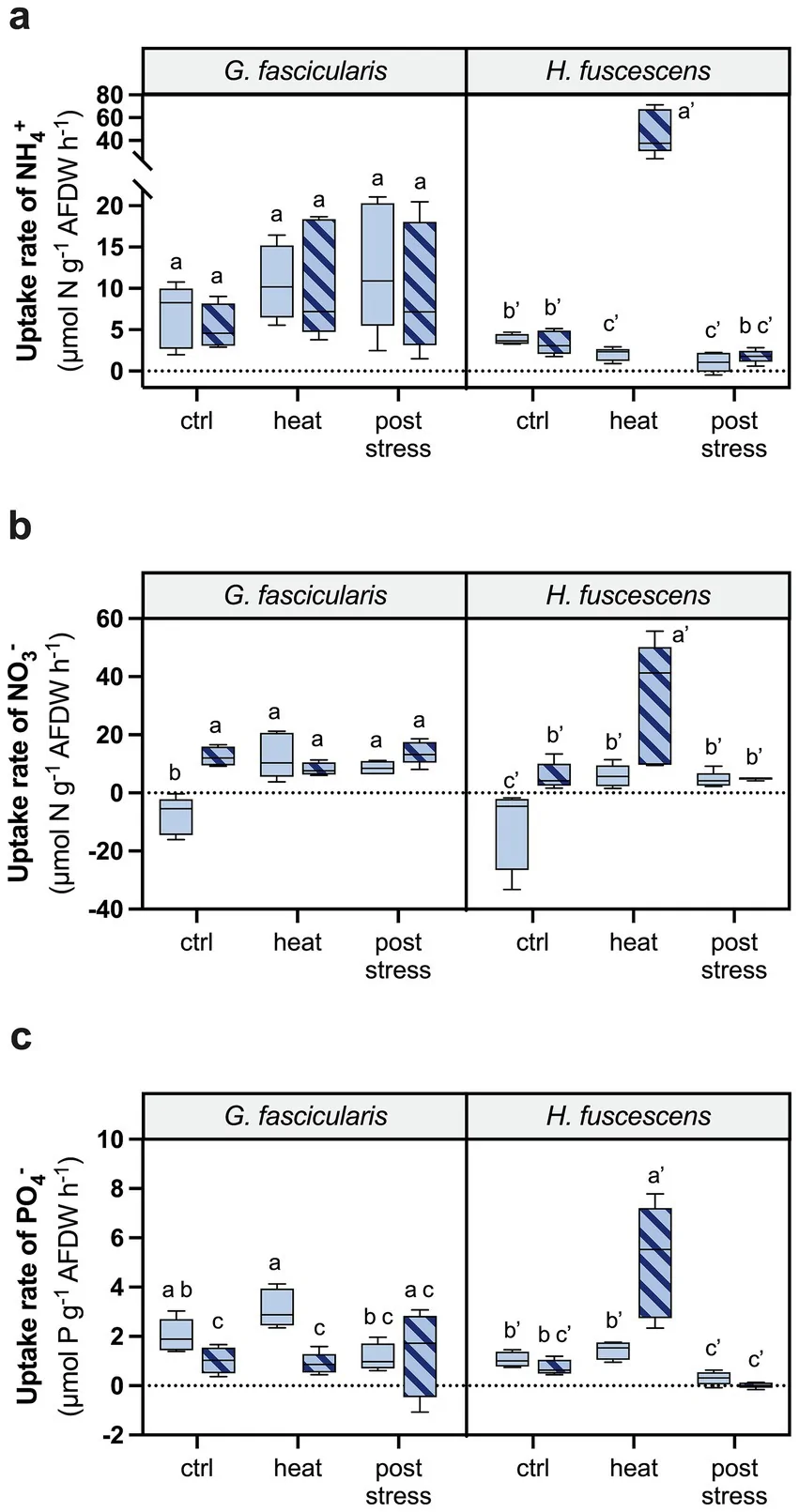
Effect of nutrient enrichment and warming on coral uptake of inorganic nutrients. Boxplots show uptake rates of **(a)** ammonium, **(b)** nitrate, and **(c)** phosphate (μmol N g^−1^ AFDW h^−1^) by *Galaxea fascicularis* and *Heteroxenia fuscescens* under different experimental conditions. Plain blue boxplots represent corals not enriched in nutrients (“C” corals) while stripped blue boxplots represent corals exposed to nutrient enrichment (“NP corals”). “T*rl*” refers to control conditions of temperature at 25 °C, “heat” refers to an exposure to 30 °C for 14 days, and “post-stress” refers to the recovery phase, with temperature going back to 25 °C for 14 days. Data shown as mean ± SD (*n* = 5 nubbins per species and condition). Letters indicate significant differences between treatments, for each species independently.

Nutrient enrichment alone did not significantly change NH₄^+^ uptake rates compared to the control condition in either species (Figure 6a). However, both *G. fascicularis* and *H. fuscescens* shifted from net NO₃^−^ release (negative fluxes) in non-enriched nubbins (“C”) to net uptake for nutrient-enriched (“NP”) nubbins (*p* < 0.001 and *p* < 0.05, respectively; Figure 6b), suggesting a strong response of nitrate fluxes to nitrate availability. In addition, enrichment reduced PO₄^3−^ uptake by 50% in *G. fascicularis* (*p* < 0.05; Figure 6c).

#### 3.2.2 Effect of heat stress

At 30 °C, both species switched from NO₃^−^ release to net uptake (*p* < 0.001 for *G. fascicularis*; *p* < 0.05 for *H. fuscescens*; Figure 6b), a response maintained during the post-stress phase (*p* < 0.01 and *p* < 0.05). In *G. fascicularis*, NH₄^+^ uptake remained stable, while PO₄^3−^ uptake decreased by 60% between heat and post-stress phase (*p* < 0.01; Figure 6c) yet remaining within control levels. In *H. fuscescens*, the increase in NO₃^−^ uptake at 30 °C was accompanied by a 50% reduction in NH₄^+^ uptake (*p* < 0.05; Figure 6a), which failed to recover during post stress phase (*p* < 0.01). In addition, PO₄^3−^ uptake sharply declined during the post stress phase (−70% to −80% compared to control and heat conditions; *p* < 0.05 and *p* < 0.001; Figure 6c).

#### 3.2.3 Combined effect of nutrient enrichment and temperature

Under combined enrichment and warming, *G. fascicularis* exhibited no major change in nutrient uptake compared to the control condition, except for significantly lower PO₄^3−^ uptake in NPT than C nubbins at 30 °C (*p* < 0.001; Figure 6c). On the contrary, *H. fuscescens* showed strong stimulation at 30 °C, with uptake rates of all nutrients tested increasing by roughly one order of magnitude (*p* < 0.05–0.01; Figures 6a–c), and NPT nubbins showing significantly higher uptake rates than C nubbins (NH₄^+^ × 23, *p* < 0.01; NO₃^−^ × 3, *p* < 0.05; PO₄^3−^ × 5, *p* < 0.01). During the post-stress phase, however, all uptake rates by *H. fuscescens* significantly decreased (*p* < 0.05) and returned to control levels, with PO₄^3−^ fluxes stabilizing near zero, indicating balanced uptake and release.

## 4 Discussion

This study shows that the two coral holobionts used very different physiological strategies when exposed to nutrient enrichment, heat stress, or both (Figure 1). *Galaxea fascicularis* was highly sensitive to individual and combined stressors, exhibiting impaired photosynthesis and carbon allocation under stress, however accompanied by increases in energy storage or tissue nutrient content. In contrast, *H. fuscescens* maintained stable or increased performance under single stressors, including increases in translocation rates and lipid content but responded negatively to the combined stress. Under such condition, it experienced rapid bleaching, altered carbon transfer, and temporary depletion in energy reserves, followed by substantial post-stress energy accumulation. Together, these findings demonstrate holobiont-specific differences in stress tolerance, symbiosis stability, carbon allocation, and energy storage dynamics, highlighting distinct short- and long-term physiological response trajectories.

### 4.1 Different carbon allocation strategies under single stressors

Our ^13^C-bicarbonate labelling experiments revealed fundamental differences in how the two holobionts managed their carbon budget under stress. In *G. fascicularis*, nutrient enrichment (NP) altered internal carbon partitioning by decoupling photosynthetic carbon fixation from host translocation, suggesting that the symbionts shifted towards a less cooperative or more self-retentive state (Lesser et al., 2013). While total carbon fixation (P_C_) remained stable, the proportion of carbon translocated to the host (T_S_) decreased from 85% of fixed carbon in control condition to 9% in nutrient enriched condition. This decline was primarily driven by a 9-fold increase in symbiont respiration (R_S_), which accounted for 86% of the total fixed carbon. Elevated R_S_ likely reflects increased metabolic maintenance and nutrient assimilation costs within the symbionts (Rädecker et al., 2021), effectively diverting carbon away from translocation. This metabolic reallocation is consistent with a transition toward a parasitic or “selfish” symbiont phenotype (Wiedenmann et al., 2013; Ezzat et al., 2015; Baker et al., 2018), despite stable symbiont densities. A comparable pattern emerged under thermal stress alone. *Galaxea fascicularis* suffered delayed bleaching accompanied by a drop in the percentage of fixed carbon translocated to the host (T_S_ 11% of P_C_) while R_S_ was much higher than in the other stress conditions (Baker et al., 2018). Together, these results suggest that both nutrient and thermal stress impair the metabolic coupling between symbiont photosynthesis and host carbon acquisition, primarily by increasing symbiont respiratory demand and thereby constraining the carbon available for host support. This shift to parasitic behaviour is characteristic of *Cladocopium* symbionts. Under stable, oligotrophic conditions, *Cladocopium* typically behaves as a highly cooperative partner: it exhibits high photosynthetic rates and transfers a substantial fraction of fixed carbon to the coral, supporting host metabolism and calcification (Stat et al., 2008; Ezzat et al., 2017). However, this cooperative state is environmentally contingent. Under thermal stress, *Cladocopium* experiences rapid photoinhibition and elevated oxidative stress, leading to declines in photosynthetic efficiency (F_v_/F_m_), carbon fixation, and translocation (Rowan et al., 1997; Warner et al., 1999; Rowan, 2004). Nutrient enrichment, particularly increased dissolved inorganic nitrogen (DIN), further shifts the position of the symbiosis along the cooperation–parasitism continuum. In *Cladocopium*-dominated associations, nitrogen enrichment often leads to increased symbiont densities and decreased host benefit, consistent with a shift toward a more parasitic phenotype (Cunning and Baker, 2013; Wiedenmann et al., 2013; Wooldridge, 2013; Baker et al., 2018).

Conversely, symbionts of *H. fuscescens* exhibited a “host-centric” strategy, maintaining high carbon translocation rates under both nutrient and thermal stress alone. In this species, although P_C_ remained stable, translocation rates actually increased (reaching up to 75% of total carbon fixed compared to 45% in control condition) while symbiont respiration decreased by two. *Durusdinium* is widely characterized as more thermally tolerant (Rowan, 2004; Berkelmans and Van Oppen, 2006). Although baseline carbon translocation and host growth rates are sometimes lower in corals dominated by *Durusdinium* compared to *Cladocopium* under optimal conditions (Little et al., 2004; Cantin et al., 2009; Jones and Berkelmans, 2010; Wall et al., 2020), *Durusdinium* maintains photosynthetic performance and carbon assimilation more effectively at elevated temperatures (Rowan, 2004). Results of this study suggest that *Durusdinium* may also exhibit more conservative growth responses under nutrient enrichment in some host–symbiont pairings, potentially maintaining more stable carbon allocation (Cunning et al., 2015), though responses can be strain- and host-specific.

### 4.2 Combined stress: balanced nutrient enrichment delays or enhances the negative effects of thermal stress depending on coral holobiont

A key finding of this study was the transient mitigation of thermal stress in *G. fascicularis* under balanced nutrient enrichment (N:P ~ 4–5, supplying both N and P). At 30 °C, nutrient enriched colonies showed enhanced photosynthetic performances and a 20-fold increase in T_S_ compared to the same treatment at 25 °C. This response supports the hypothesis that phosphorus availability plays a critical role in maintaining thylakoid membrane integrity and photosynthetic functions during heat stress (Wiedenmann et al., 2013; Ezzat et al., 2016a; Rosset et al., 2017). However, this apparent physiological “buffer” was short-lived. It could not prevent significant changes in tissue stoichiometry (lower C:P and higher C:N ratios) and a downward trend in symbiont density (two times lower than control levels, although not statistically significant) during the post-stress phase, suggesting that nutrient enrichment may only delay, rather than prevent, physiological collapse under heat stress. This is in agreement with the findings of Hidaka and Miyagi (1999) who demonstrated that N or P enrichment was insufficient to prevent bleaching in *G. fascicularis* at 32 °C.

Paradoxically, the same nutrient regime that transiently buffered thermal stress in *G. fascicularis* triggered immediate and severe bleaching in *H. fuscescens*, despite this octocoral showing resilience to each stressor in isolation. This contrasting response may reflect a more aggressive nutrient acquisition strategy in *H. fuscescens*, which increased its uptake rates of NH_4_^+^, NO_3_^−^ and PO_4_^3−^ at 30 °C. Notably, the massive 23-fold increase in NH_4_^+^ uptake under NPT suggests an internal “nutrient overload” or toxic accumulation that could have triggered symbiont expulsion (Béraud et al., 2013; Wiedenmann et al., 2013). Importantly, our elemental analyses were conducted at the holobiont tissue level and therefore may mask compartment-specific shifts between host and symbionts. It is conceivable that nitrogen uptake far exceeded phosphorus assimilation internally (even though nutrients were supplied at a balanced external ratio) leading to a severe intracellular N:P imbalance within the symbionts. This scenario could have induced functional phosphorus limitation at the cellular level, constraining phospholipid synthesis and membrane repair processes. Under elevated temperature, such P-starvation would render thylakoid membranes particularly vulnerable to oxidative damage and thermal destabilization (Wiedenmann et al., 2013; Ezzat et al., 2016a), accelerating photo-physiological collapse and symbiosis breakdown. In contrast, nutrient uptake rates in *G. fascicularis* were unaffected by the nutrient and temperature conditions. Taken together, these results suggest that species-specific nutrient uptake strategies may dictate whether enrichment acts as a buffer or a physiological toxin when coupled to thermal stress.

### 4.3 Metabolic depression and energy reallocation as a survival mechanism

A striking result of this study is the significant “energy savings” observed in *H. fuscescens* during the post-stress phase following combined nutrient enrichment and thermal stress (NPT). Despite a severe reduction in symbiont density and a 3-fold decrease in total carbon fixation (P_C_), the holobiont nearly doubled its total tissue energy content, driven by a massive accumulation of carbohydrates (+70%) and lipids (+125%). In the absence of significant dissolved organic matter (DOM) or heterotrophic feeding opportunities in our experimental setup, this accumulation likely results from a fundamental internal reallocation of the holobiont’s carbon budget rather than increased heterotrophic acquisition.

We propose that this energy storage reflects a survival strategy where organisms navigate environmental stress by drastically reducing energy expenditure (Sokolova et al., 2012). This “shutdown” is evidenced by the respiration rates, which were three times lower in the post-stress phase than in control conditions. Such a metabolic shift creates a transient production-consumption imbalance: although autotrophic carbon production is reduced, the energy demand for maintenance, growth, and activity drops even more sharply. Consequently, fixed carbon that would normally be respired is instead diverted into macromolecular stores, resulting in the observed lipid and carbohydrate surplus. In *H. fuscescens*, this metabolic depression is likely achieved through the suspension of energetically expensive physiological functions. For Xeniids, the active pulsation of polyp tentacles is a primary energy sink, consuming up to 50% of the daily budget (Kremien et al., 2013). Previous studies have shown that *Xenia* species reduce or entirely cease pulsing under stress as a conservation measure (Thobor et al., 2022; Mezger et al., 2025). By “turning off” pulsing and likely growth (functions that were not measured here but are typically suppressed under stress), *H. fuscescens* prioritizes long-term survival by redirecting remaining photosynthates toward energy reserves. These results align with recent findings by Pupier et al. (2024), who demonstrated that *H. fuscescens* maintains higher carbohydrate and lipid levels than scleractinian corals (e.g., *Stylophora pistillata* in symbiosis with clade *Symbiodinium*) when facing thermal stress.

While *G. fascicularis* also displayed some capacity for post-heat energy accumulation, it lacked the robust recovery and metabolic plasticity exhibited by the octocoral under combined stressors. This ability to rapidly enter a “storage-mode” metabolism provides a vital physiological buffer that may explain the increasing competitive success of octocoral holobionts over scleractinians on anthropogenically impacted and warming reefs (Norström et al., 2009; Baum et al., 2016; Reverter et al., 2022; Lalas et al., 2024). Ultimately, our findings highlight that energy reserve measurements serve as a powerful indicator of resilience and stress legacy, revealing critical physiological trade-offs that remain hidden when relying solely on photosynthetic metrics.

## 5 Conclusion and ecological implications

This study shows that key mechanisms underlying coral resilience emerge from the complex interaction between local nutrient availability and global thermal stress. Our findings reveal that these interactions are highly holobiont-specific and time-dependent, dictated by distinct carbon allocation strategies during both the stress and recovery phases. While the scleractinian *G. fascicularis* (associated with *Cladocopium*) is initially more sensitive to individual stressors, marked by a sharp drop in carbon translocation driven by elevated symbiont respiration, its physiological collapse under heat can be temporarily buffered by a balanced nutrient supply. In contrast, the octocoral *H. fuscescens* (associated with *Durusdinium*) exhibits greater physiological tolerance to single-stressor conditions but reaches a critical threshold under the synergistic “overdose” of combined stress. However, its ability to rapidly restore host energy reserves during recovery suggests a greater capacity for short-term physiological recovery following stress.

Ultimately, our results emphasize that coral resilience cannot be accurately predicted from photosynthetic performance alone. Instead, it emerges from a holobiont’s ability to dynamically regulate its carbon budget and maintain metabolic balance. A central discovery of this work is the “energy-saving” strategy observed following stress exposure, where both species significantly increased their lipid and carbohydrate reserves. For *H. fuscescens*, this “conservation-based” strategy of prioritizing host energy stores may provide the metabolic plasticity required to survive the increasing frequency of compound stress events. As reefs transition under global change, future community composition may increasingly favor coral–symbiont pairings capable of efficiently regulating energy storage and carbon allocation under fluctuating environmental conditions. Future research should prioritize investigating how functional diversity dictates these energetic strategies, as this will be essential for predicting the future functional integrity of coral reef ecosystems.

## Data Availability

The original contributions presented in the study are included in the article/Supplementary material, further inquiries can be directed to the corresponding author.
