# Supplementary material for: Energetic and carbon allocation strategies shape coral holobiont responses to combined thermal and nutrient stress
Source: Front Microbiol. 2026 Aug 10;17:1909606. doi: 10.3389/fmicb.2026.1909606 (PMC13500517; doi:10.3389/fmicb.2026.1909606)
Supplement: Supplementary file 1 [file Data_Sheet_1.DOCX]

**Supplementary Information**

**Material and Methods**

**Calculations for the carbon budget** (from Tremblay et al., 2012)

Carbon incorporation rates (µg C g⁻^1^ AFDW h⁻^1^) in the symbionts (ρ_S_) and coral host tissue (ρ_H_) were calculated as follow:

$$\rho=\frac{(C_{meas}-C_{nat})\times M_{sample}\times M_{C}}{(C_{inc}-C_{meas})\times(t_{pulse}+t_{chase})\times AFDW}$$

where C_meas_ and C_nat_ are the percentages of ^13^C measured on the samples (symbionts or host tissues) from ^13^C -enriched experiments and controls, respectively. C_inc_ is the percent ^13^C enrichment of the incubation medium; M_sample_ is the mass of the freeze-dried sample (mg); M_C_ is the mass of carbon per milligram of symbiont or host tissue (μg mg^-1^); AFDW is the ash- free dry weight (g); and *t*_pulse_ and *t*_chase_ are incubation times (h) of the nubbins in the enriched and non-enriched incubation media, respectively. C_inc_ varied during the pulse chase and was calculated as:

$$C_{inc}=\frac{\left( C_{pulse}\times t_{pulse} \right)+(C_{chase}\times t_{chase})}{(t_{pulse}+t_{chase})}$$

where C_pulse_ and C_chase_ are the percent ^13^C enrichment of the enriched and non-enriched incubation media, respectively (C_chase_ = 1.0827%).

The amount of carbon acquired through photosynthesis, translocated from the symbionts to the host (T_S_, µg C g⁻^1^ AFDW h⁻^1^) was calculated as:

$$T_{S}=P_{C}-\rho_{S}-R_{S}$$

where P_C_ is the total amount of autotrophic carbon produced by the symbionts; ρ_S_ is the

carbon assimilation rate and R_S_ the respiration rate of the symbionts. The percentage of translocated carbon was obtained by dividing T_S_ by P_C_, and multiplying the result by 100.

Finally, the amount of carbon lost (C_L_) through respiration (R_S_ and R_H_) and through particulate and dissolved carbon release (C_excret_) is:

$$C_{L}=P_{C}-\rho_{S}-\rho_{H}$$

The percentage of carbon lost is obtained by dividing C_L_ by P_C_, and multiplying the result by 100.

**Supplementary figures**

**Fig. S1. Effect of nutrient enrichment and warming on coral oxygen fluxes.**

Boxplots show (a) net photosynthesis, (b) gross photosynthesis and (c) respiration rates (µmol O_2_ g^-1^ AFDW h^-1^) for *Galaxea fascicularis* and *Heteroxenia fuscescens* under different experimental conditions. Plain blue boxplots represent corals not enriched in nutrients (“C” corals) while stripped blue boxplots represent corals exposed nutrient enrichments (“NP corals”). “*ctrl*” refers to control conditions of temperature at 25°C, “heat” refers to an exposure to 30°C for 14 days, and “post-stress” refers to the recovery phase, with temperature going back to 25°C for 14 days. Data shown as mean ± SD (n = 5 nubbins per species and condition). Letters indicate significant differences between treatments, for each species independently.

**Fig. S2. Effect of nutrient enrichment and warming on coral carbon budget.**

Boxplots show (a) carbon fixation by photosynthesis (µg C g^-1^ AFDW h^-1^), (b) incorporation rates of carbon in symbionts (µg C g^-1^ AFDW h^-1^), (c) symbionts respiration (µmol O_2_ g^-1^ AFDW h^-1^), (d) incorporation rates of carbon in host tissue (µg C g^-1^ AFDW h^-1^), translocation rates of carbon from the symbionts to the host (e, µg C g^-1^ AFDW h^-1^; and f, relative percentage of carbon fixed translocated), carbon loss (g, µg C g^-1^ AFDW h^-1^; and h, relative percentage of carbon fixed translocated), for *Galaxea fascicularis* and *Heteroxenia fuscescens* under different experimental conditions. Plain blue boxplots represent corals not enriched in nutrients (“C” corals) while stripped blue boxplots represent corals exposed nutrient enrichments (“NP corals”). “*ctrl*” refers to control conditions of temperature at 25°C, “heat” refers to an exposure to 30°C for 14 days, and “post-stress” refers to the recovery phase, with temperature going back to 25°C for 14 days. Data shown as mean ± SD (n = 5 nubbins per species and condition). Letters indicate significant differences between treatments, for each species independently.

**Fig. S3. Effect of nutrient enrichment and warming on coral elemental composition.**

Boxplots show (a) carbon, (b) nitrogen and (c) phosphorus tissue content (mg g^-1^ AFDW), as well as (d) C:N, (e) C:P and (f) N:P ratios in tissue, for *Galaxea fascicularis* and *Heteroxenia fuscescens* under different experimental conditions. Plain blue boxplots represent corals not enriched in nutrients (“C” corals) while stripped blue boxplots represent corals exposed nutrient enrichments (“NP corals”). “*ctrl*” refers to control conditions of temperature at 25°C, “heat” refers to an exposure to 30°C for 14 days, and “post-stress” refers to the recovery phase, with temperature going back to 25°C for 14 days. Data shown as mean ± SD (n = 5 nubbins per species and condition). Letters indicate significant differences between treatments, for each species independently.

**Fig. S4. Data normalized to symbiont density.**

Boxplots show (a) net photosynthesis, (b) gross photosynthesis and (c) respiration rates (µmol O_2_ 10^-7^ symbiont cells h^-1^), uptake rates of (d) ammonium, (e) nitrate and (f) phosphate (µmol N 10^-7^ symbiont cells h^-1^), and (g) incorporation rates of carbon in symbionts and (h) translocation rates to the host (µg C 10^-7^ symbiont cells h^-1^), for *Galaxea fascicularis* and *Heteroxenia fuscescens* under different experimental conditions. Plain blue boxplots represent corals not enriched in nutrients (“C” corals) while stripped blue boxplots represent corals exposed nutrient enrichments (“NP corals”). “*ctrl*” refers to control conditions of temperature at 25°C, “heat” refers to an exposure to 30°C for 14 days, and “post-stress” refers to the recovery phase, with temperature going back to 25°C for 14 days. Data shown as mean ± SD. Letters indicate significant differences between treatments, for each species independently.

**Supplementary tables**

**Table S1.** **Statistical analysis and results of the effects of nutrient enrichment and warming on coral physiology.** “C”, control, non-enriched nubbins; “NP”, nutrient-enriched nubbins; “*ctrl*”, control temperature at 25°C; “heat”, short-term heat stress at 30°C for 14 days; “recov”, recovery phase, with temperature going back to 25°C for 14 days. Significant differences are displayed in bold.

|  | **Symbiont density (Fig. 1a)** | | | | | | | |  |
| --- | --- | --- | --- | --- | --- | --- | --- | --- | --- |
| **Kruskal - Wallis** | Grouped by | | variable | | n | statistic | df | p |  |
|  | ctrl | C | Species | | 10 | 5.77 | 1 | **0.016** |  |
|  |  | NP |  |  | 10 | 6.82 | 1 | **0.009** |  |
|  | heat | C |  |  | 10 | 6.82 | 1 | **0.009** |  |
|  |  | NP |  |  | 10 | 6.82 | 1 | **0.009** |  |
|  | recov | C |  |  | 8 | 4.50 | 1 | **0.034** |  |
|  |  | NP |  |  | 8 | 5.33 | 1 | **0.021** |  |
|  | Galaxea | C | Temperature | | 14 | 8.46 | 2 | **0.015** |  |
|  |  | NP |  |  | 14 | 7.63 | 2 | **0.022** |  |
|  | Heterox | C |  |  | 14 | 2.85 | 2 | 0.241 |  |
|  |  | NP |  |  | 14 | 5.48 | 2 | 0.065 |  |
|  | Galaxea | ctrl | Nutrient condition | | 10 | 1.32 | 1 | 0.251 |  |
|  |  | heat |  |  | 10 | 1.84 | 1 | 0.175 |  |
|  |  | recov |  |  | 8 | 4.08 | 1 | **0.043** |  |
|  | Heterox | ctrl |  |  | 10 | 1.32 | 1 | 0.251 |  |
|  |  | heat |  |  | 10 | 1.84 | 1 | **0.028** |  |
|  |  | recov |  |  | 8 | 4.50 | 1 | **0.034** |  |
| **Dunn** |  | | | Pairwise comparison | statistic | p | p.adj | p.adj.signif |  |
|  | Species | ctrl | C | Gal-Heter | 2.40 | 0.016 | **0.016** | ***** |  |
|  |  |  | NP | Gal-Heter | 2.61 | 0.009 | **0.009** | ****** |  |
|  |  | heat | C | Gal-Heter | 2.61 | 0.009 | **0.009** | ****** |  |
|  |  |  | NP | Gal-Heter | 2.61 | 0.009 | **0.009** | ****** |  |
|  |  | recov | C | Gal-Heter | 2.12 | 0.034 | **0.034** | ***** |  |
|  |  |  | NP | Gal-Heter | 2.31 | 0.021 | **0.021** | ***** |  |
|  | Temperature | Galaxea | C | ctrl-heat | -0.68 | 0.496 | 0.496 | ns |  |
|  |  |  |  | ctrl-recov | -2.82 | 0.005 | **0.015** | ***** |  |
|  |  |  |  | heat-recov | -2.17 | 0.030 | **0.049** | ***** |  |
|  |  |  | NP | ctrl-heat | 1.36 | 0.174 | 0.278 | ns |  |
|  |  |  |  | ctrl-recov | -1.48 | 0.139 | 0.278 | ns |  |
|  |  |  |  | heat-recov | -2.76 | 0.006 | **0.017** | ***** |  |
|  |  | Heterox | C | ctrl-heat | 0.73 | 0.465 | 0.583 | ns |  |
|  |  |  |  | ctrl-recov | 1.69 | 0.091 | 0.274 | ns |  |
|  |  |  |  | heat-recov | 1.05 | 0.292 | 0.583 | ns |  |
|  |  |  | NP | ctrl-heat | -1.97 | 0.049 | 0.121 | **ns** |  |
|  |  |  |  | ctrl-recov | -2.05 | 0.040 | 0.121 | **ns** |  |
|  |  |  |  | heat-recov | -0.20 | 0.845 | 0.845 | ns |  |
|  | Nutrient condition | Galaxea | ctrl | C-NP | -1.15 | 0.251 | 0.251 | ns |  |
|  |  |  | heat | C-NP | 1.36 | 0.175 | 0.175 | ns |  |
|  |  |  | recov | C-NP | 2.02 | 0.043 | **0.043** | ***** |  |
|  |  | Heterox | ctrl | C-NP | 1.15 | 0.251 | 0.251 | ns |  |
|  |  |  | heat | C-NP | -1.36 | 0.028 | **0.028** | ***** |  |
|  |  |  | recov | C-NP | -2.12 | 0.034 | **0.034** | ***** |  |
|  | **Chlorophyll content per symbiont (Fig. 1b)** | | | | | | | |  |
| **Kruskal - Wallis** | Grouped by | | variable | | n | statistic | df | p |  |
|  | ctrl | C | Species | | 10 | 0.88 | 1 | 0.347 |  |
|  |  | NP |  |  | 10 | 0.24 | 1 | 0.624 |  |
|  | heat | C |  |  | 10 | 6.82 | 1 | **0.009** |  |
|  |  | NP |  |  | 10 | 0.01 | 1 | 0.917 |  |
|  | recov | C |  |  | 8 | 4.50 | 1 | **0.034** |  |
|  |  | NP |  |  | 8 | 2.08 | 1 | 0.149 |  |
|  | Galaxea | C | Temperature | | 14 | 11.57 | 2 | **0.003** |  |
|  |  | NP |  |  | 14 | 10.19 | 2 | **0.006** |  |
|  | Heterox | C |  |  | 14 | 6.02 | 2 | **0.049** |  |
|  |  | NP |  |  | 14 | 9.41 | 2 | **0.009** |  |
|  | Galaxea | ctrl | Nutrient condition | | 10 | 1.50 | 1 | 0.221 |  |
|  |  | heat |  |  | 10 | 1.32 | 1 | 0.251 |  |
|  |  | recov |  |  | 8 | 4.08 | 1 | **0.043** |  |
|  | Heterox | ctrl |  |  | 10 | 0.53 | 1 | 0.465 |  |
|  |  | heat |  |  | 10 | 6.82 | 1 | **0.009** |  |
|  |  | recov |  |  | 8 | 3.13 | 1 | 0.077 |  |
| **Dunn** |  | | | Pairwise comparison | statistic | p | p.adj | p.adj.signif |  |
|  | Species | ctrl | C | Gal-Heter | -0.94 | 0.347 | 0.347 | ns |  |
|  |  |  | NP | Gal-Heter | -0.49 | 0.624 | 0.624 | ns |  |
|  |  | heat | C | Gal-Heter | 2.61 | 0.009 | **0.009** | ****** |  |
|  |  |  | NP | Gal-Heter | 0.10 | 0.917 | 0.917 | ns |  |
|  |  | recov | C | Gal-Heter | -2.12 | 0.034 | **0.034** | ***** |  |
|  |  |  | NP | Gal-Heter | -1.44 | 0.149 | 0.149 | ns |  |
|  | Temperature | Galaxea | C | ctrl-heat | -1.89 | 0.059 | 0.118 | ns |  |
|  |  |  |  | ctrl-recov | 1.60 | 0.001 | **0.001** | ****** |  |
|  |  |  |  | heat-recov | 3.39 | 0.001 | **0.002** | ****** |  |
|  |  |  | NP | ctrl-heat | -1.82 | 0.069 | 0.138 | ns |  |
|  |  |  |  | ctrl-recov | 1.27 | 0.204 | 0.204 | ns |  |
|  |  |  |  | heat-recov | 3.16 | 0.002 | **0.005** | ****** |  |
|  |  | Heterox | C | ctrl-heat | -0.65 | 0.516 | 0.516 | ns |  |
|  |  |  |  | ctrl-recov | 1.85 | 0.026 | **0.026** | ***** |  |
|  |  |  |  | heat-recov | 2.41 | 0.016 | **0.047** | ***** |  |
|  |  |  | NP | ctrl-heat | -2.34 | 0.019 | **0.038** | ***** |  |
|  |  |  |  | ctrl-recov | 0.64 | 0.521 | 0.521 | ns |  |
|  |  |  |  | heat-recov | 2.85 | 0.004 | **0.013** | ***** |  |
|  | Nutrient condition | Galaxea | ctrl | C-NP | -1.22 | 0.221 | 0.221 | ns |  |
|  |  |  | heat | C-NP | -1.15 | 0.251 | 0.251 | ns |  |
|  |  |  | recov | C-NP | -2.02 | 0.043 | **0.043** | ***** |  |
|  |  | Heterox | ctrl | C-NP | -0.73 | 0.465 | 0.465 | ns |  |
|  |  |  | heat | C-NP | -2.61 | 0.009 | **0.009** | ****** |  |
|  |  |  | recov | C-NP | -1.77 | 0.077 | 0.077 | ns |  |
|  | **Chlorophyll per AFDW (Fig. 1c)** | | | | | | | | |
| **3-way ANOVA** | Effect | | | DFn | DFd | F | p | p<.05 | ges |
|  | Species | | | 1 | 43 | 110.7 | **1.81E-13** | ***** | 0.72 |
|  | Temperature | | | 2 | 43 | 5.6 | **7.00E-03** | ***** | 0.207 |
|  | Condition | | | 1 | 43 | 9.6 | **3.00E-03** | ***** | 0.183 |
|  | Species:Temperature | | | 2 | 43 | 4.3 | **2.00E-02** | ***** | 0.166 |
|  | Species:Condition | | | 1 | 43 | 9.0 | **4.00E-03** | ***** | 0.173 |
|  | Temperature:Condition | | | 2 | 43 | 11.5 | **1.02E-04** | ***** | 0.348 |
|  | Species:Temperature:Condition | | | 2 | 43 | 12.4 | **5.51E-05** | ***** | 0.366 |
| **Tukey** | Variable | | | Pairwise comparison | estimate | conf.low | conf.high | p.adj | p.adj.signif |
|  | Nutrient condition | Galaxea | ctrl | C-NP | -0.04 | -0.21 | 0.13 | 0.617 | ns |
|  |  |  | heat | C-NP | 0.00 | -0.06 | 0.06 | 0.944 | ns |
|  |  |  | recov | C-NP | -0.01 | -0.07 | 0.06 | 0.809 | ns |
|  |  | Heterox | ctrl | C-NP | 0.51 | -0.36 | 1.38 | 0.216 | ns |
|  |  |  | heat | C-NP | -0.82 | -1.23 | -0.41 | **0.002** | ****** |
|  |  |  | recov | C-NP | -1.44 | -1.99 | -0.88 | **0.001** | ******* |
|  | Temperature | Galaxea | C | ctrl-heat | -0.13 | -0.28 | 0.02 | 0.101 | ns |
|  |  |  |  | ctrl-recov | -0.11 | -0.27 | 0.05 | 0.184 | ns |
|  |  |  |  | heat-recov | 0.01 | -0.15 | 0.17 | 0.967 | ns |
|  |  |  | NP | ctrl-heat | -0.09 | -0.20 | 0.02 | 0.113 | ns |
|  |  |  |  | ctrl-recov | -0.08 | -0.20 | 0.04 | 0.195 | ns |
|  |  |  |  | heat-recov | 0.01 | -0.11 | 0.13 | 0.974 | ns |
|  |  | Heterox | C | ctrl-heat | 0.16 | -0.45 | 0.77 | 0.757 | ns |
|  |  |  |  | ctrl-recov | 1.09 | 0.44 | 1.74 | **0.002** | ****** |
|  |  |  |  | heat-recov | 0.93 | 0.28 | 1.58 | **0.007** | ****** |
|  |  |  | NP | ctrl-heat | -1.16 | -2.08 | -0.24 | **0.016** | ***** |
|  |  |  |  | ctrl-recov | -0.85 | -1.78 | 0.07 | 0.071 | ns |
|  |  |  |  | heat-recov | 0.31 | -0.66 | 1.28 | 0.670 | ns |
|  | Species | ctrl | C | Gal-Heter | 0.66 | 0.24 | 1.09 | **0.007** | ****** |
|  |  |  | NP | Gal-Heter | 1.21 | 0.43 | 1.99 | **0.007** | ****** |
|  |  | heat | C | Gal-Heter | 0.95 | 0.61 | 1.29 | **0.000** | ******* |
|  |  |  | NP | Gal-Heter | 0.14 | 0.02 | 0.26 | **0.031** | ***** |
|  |  | recov | C | Gal-Heter | 1.87 | 1.43 | 2.30 | **0.000** | ******** |
|  |  |  | NP | Gal-Heter | 0.44 | 0.09 | 0.79 | **0.023** | ***** |
|  | **P:R ratio (Fig. 1d)** | | | | | | | | |
| **3-way ANOVA** | Effect | | | DFn | DFd | F | p | p<.05 | ges |
|  | Species | | | 1 | 41 | 2.55 | 0.118 |  | 0.059 |
|  | Temperature | | | 2 | 41 | 0.04 | 0.964 |  | 0.002 |
|  | Condition | | | 1 | 41 | 6.55 | **0.014** | ***** | 0.138 |
|  | Species:Temperature | | | 2 | 41 | 1.79 | 0.179 |  | 0.08 |
|  | Species:Condition | | | 1 | 41 | 0.66 | 0.421 |  | 0.016 |
|  | Temperature:Condition | | | 2 | 41 | 8.97 | **0.001** | ***** | 0.304 |
|  | Species:Temperature:Condition | | | 2 | 41 | 1.07 | 0.354 |  | 0.049 |
| **Tukey** | Variable | | | Pairwise comparison | estimate | conf.low | conf.high | p.adj | p.adj.signif |
|  | Nutrient condition | Galaxea | ctrl | C-NP | -0.39 | -0.59 | -0.20 | **0.002** | ****** |
|  |  |  | heat | C-NP | 0.44 | 0.34 | 0.55 | **0.000** | ******** |
|  |  |  | recov | C-NP | 0.24 | -0.80 | 1.29 | 0.574 | ns |
|  |  | Heterox | ctrl | C-NP | -0.05 | -0.28 | 0.17 | 0.593 | ns |
|  |  |  | heat | C-NP | 0.35 | 0.07 | 0.62 | **0.019** | ***** |
|  |  |  | recov | C-NP | 0.34 | 0.04 | 0.64 | **0.034** | ***** |
|  | Temperature | Galaxea | C | ctrl-heat | -0.48 | -0.84 | -0.13 | **0.011** | ***** |
|  |  |  |  | ctrl-recov | -0.24 | -0.62 | 0.13 | 0.229 | ns |
|  |  |  |  | heat-recov | 0.24 | -0.12 | 0.60 | 0.209 | ns |
|  |  |  | NP | ctrl-heat | 0.35 | -0.32 | 1.02 | 0.349 | ns |
|  |  |  |  | ctrl-recov | 0.39 | -0.37 | 1.16 | 0.363 | ns |
|  |  |  |  | heat-recov | 0.04 | -0.69 | 0.77 | 0.987 | ns |
|  |  | Heterox | C | ctrl-heat | -0.10 | -0.36 | 0.16 | 0.559 | ns |
|  |  |  |  | ctrl-recov | -0.25 | -0.53 | 0.02 | 0.072 | ns |
|  |  |  |  | heat-recov | -0.15 | -0.43 | 0.12 | 0.331 | ns |
|  |  |  | NP | ctrl-heat | 0.30 | -0.02 | 0.62 | 0.071 | ns |
|  |  |  |  | ctrl-recov | 0.14 | -0.20 | 0.48 | 0.542 | ns |
|  |  |  |  | heat-recov | -0.16 | -0.50 | 0.18 | 0.441 | ns |
|  | Species | ctrl | C | Gal-Heter | -0.30 | -0.49 | -0.10 | **0.010** | ****** |
|  |  |  | NP | Gal-Heter | 0.04 | -0.19 | 0.28 | 0.664 | ns |
|  |  | heat | C | Gal-Heter | 0.09 | -0.08 | 0.25 | 0.250 | ns |
|  |  |  | NP | Gal-Heter | -0.01 | -0.25 | 0.23 | 0.920 | ns |
|  |  | recov | C | Gal-Heter | -0.30 | -0.76 | 0.16 | 0.157 | ns |
|  |  |  | NP | Gal-Heter | -0.21 | -1.16 | 0.74 | 0.592 | ns |
|  | **Net photosynthesis (Fig. S1a)** | | | | | | | |  |
| **Kruskal - Wallis** | Grouped by | | variable | | n | statistic | df | p |  |
|  | ctrl | C | Species | | 10 | 0.10 | 1 | 0.754 |  |
|  |  | NP |  |  | 10 | 2.94 | 1 | 0.086 |  |
|  | heat | C |  |  | 10 | 6.82 | 1 | **0.009** |  |
|  |  | NP |  |  | 10 | 0.10 | 1 | 0.754 |  |
|  | recov | C |  |  | 8 | 3.00 | 1 | 0.083 |  |
|  |  | NP |  |  | 8 | 4.50 | 1 | **0.034** |  |
|  | Galaxea | C | Temperature | | 14 | 7.77 | 2 | **0.021** |  |
|  |  | NP |  |  | 14 | 8.79 | 2 | **0.012** |  |
|  | Heterox | C |  |  | 14 | 3.97 | 2 | 0.137 |  |
|  |  | NP |  |  | 14 | 4.50 | 2 | 0.105 |  |
|  | Galaxea | ctrl | Nutrient condition | | 10 | 2.16 | 1 | 0.142 |  |
|  |  | heat |  |  | 10 | 6.82 | 1 | **0.009** |  |
|  |  | recov |  |  | 8 | 0.50 | 1 | 0.480 |  |
|  | Heterox | ctrl |  |  | 10 | 0.88 | 1 | 0.347 |  |
|  |  | heat |  |  | 10 | 3.15 | 1 | 0.076 |  |
|  |  | recov |  |  | 8 | 0.75 | 1 | 0.386 |  |
| **Dunn** |  | | | Pairwise comparison | statistic | p | p.adj | p.adj.signif |  |
|  | Species | ctrl | C | Gal-Heter | 0.31 | 0.754 | 0.754 | ns |  |
|  |  |  | NP | Gal-Heter | 1.71 | 0.086 | 0.086 | ns |  |
|  |  | heat | C | Gal-Heter | 2.61 | 0.009 | **0.009** | ****** |  |
|  |  |  | NP | Gal-Heter | -0.31 | 0.754 | 0.754 | ns |  |
|  |  | recov | C | Gal-Heter | 1.73 | 0.083 | 0.083 | ns |  |
|  |  |  | NP | Gal-Heter | 2.12 | 0.034 | **0.034** | ***** |  |
|  | Temperature | Galaxea | C | ctrl-heat | -1.13 | 0.257 | 0.257 | ns |  |
|  |  |  |  | ctrl-recov | -2.78 | 0.005 | **0.016** | ***** |  |
|  |  |  |  | heat-recov | -1.71 | 0.087 | 0.174 | ns |  |
|  |  |  | NP | ctrl-heat | 2.07 | 0.000 | **0.000** | ******* |  |
|  |  |  |  | ctrl-recov | -0.85 | 0.397 | 0.397 | ns |  |
|  |  |  |  | heat-recov | -2.79 | 0.005 | **0.016** | ***** |  |
|  |  | Heterox | C | ctrl-heat | -0.23 | 0.821 | 0.821 | ns |  |
|  |  |  |  | ctrl-recov | -1.85 | 0.064 | 0.192 | ns |  |
|  |  |  |  | heat-recov | -1.64 | 0.101 | 0.202 | ns |  |
|  |  |  | NP | ctrl-heat | 0.00 | 1.000 | 1.000 | ns |  |
|  |  |  |  | ctrl-recov | -1.87 | 0.061 | 0.184 | ns |  |
|  |  |  |  | heat-recov | -1.87 | 0.061 | 0.184 | ns |  |
|  | Nutrient condition | Galaxea | ctrl | C-NP | -1.47 | 0.142 | 0.142 | ns |  |
|  |  |  | heat | C-NP | 2.61 | 0.009 | **0.009** | ****** |  |
|  |  |  | recov | C-NP | 0.71 | 0.480 | 0.480 | ns |  |
|  |  | Heterox | ctrl | C-NP | 0.94 | 0.347 | 0.347 | ns |  |
|  |  |  | heat | C-NP | 1.78 | 0.076 | 0.076 | ns |  |
|  |  |  | recov | C-NP | 0.87 | 0.386 | 0.386 | ns |  |
|  | **Gross photosynthesis (Fig. S1b)** | | | | | | | | |
| **3-way ANOVA** | Effect | | | DFn | DFd | F | p | p<.05 | ges |
|  | Species | | | 1 | 42 | 18.46 | **1.01E-04** | ***** | 0.305 |
|  | Temperature | | | 2 | 42 | 6.78 | **0.003** | ***** | 0.244 |
|  | Condition | | | 1 | 42 | 1.42 | 0.241 |  | 0.033 |
|  | Species:Temperature | | | 2 | 42 | 2.28 | 0.115 |  | 0.098 |
|  | Species:Condition | | | 1 | 42 | 0.17 | 0.684 |  | 0.004 |
|  | Temperature:Condition | | | 2 | 42 | 1.42 | 0.253 |  | 0.063 |
|  | Species:Temperature:Condition | | | 2 | 42 | 4.02 | **0.025** | ***** | 0.161 |
| **Tukey** | Variable | | | Pairwise comparison | estimate | conf.low | conf.high | p.adj | p.adj.signif |
|  | Nutrient condition | Galaxea | ctrl | C-NP | -34.6 | -99.5 | 30.4 | 0.249 | ns |
|  |  |  | heat | C-NP | 69.2 | 20.9 | 117.6 | **0.011** | ***** |
|  |  |  | recov | C-NP | 3.1 | -25.1 | 31.3 | 0.790 | ns |
|  |  | Heterox | ctrl | C-NP | 136.6 | -68.9 | 342.2 | 0.164 | ns |
|  |  |  | heat | C-NP | -4.9 | -106.6 | 96.8 | 0.914 | ns |
|  |  |  | recov | C-NP | -55.0 | -119.2 | 9.3 | **0.041** | ***** |
|  | Temperature | Galaxea | C | ctrl-heat | -10.3 | -66.9 | 46.3 | 0.877 | ns |
|  |  |  |  | ctrl-recov | -76.6 | -136.7 | -16.6 | **0.014** | ***** |
|  |  |  |  | heat-recov | -66.4 | -126.4 | -6.4 | **0.031** | ***** |
|  |  |  | NP | ctrl-heat | 93.5 | 32.7 | 154.3 | **0.005** | ****** |
|  |  |  |  | ctrl-recov | -39.0 | -108.2 | 30.2 | 0.305 | ns |
|  |  |  |  | heat-recov | -132.5 | -198.7 | -66.3 | **0.001** | ******* |
|  |  | Heterox | C | ctrl-heat | 6.9 | -112.0 | 125.9 | 0.986 | ns |
|  |  |  |  | ctrl-recov | -18.5 | -144.7 | 107.7 | 0.918 | ns |
|  |  |  |  | heat-recov | -25.4 | -151.6 | 100.8 | 0.851 | ns |
|  |  |  | NP | ctrl-heat | -134.6 | -335.9 | 66.6 | 0.212 | ns |
|  |  |  |  | ctrl-recov | -210.1 | -423.6 | 3.3 | 0.054 | ns |
|  |  |  |  | heat-recov | -75.5 | -288.9 | 137.9 | 0.618 | ns |
|  | Species | ctrl | C | Gal-Heter | 56.7 | -32.2 | 145.5 | 0.180 | ns |
|  |  |  | NP | Gal-Heter | 227.9 | 2.9 | 452.9 | **0.048** | ***** |
|  |  | heat | C | Gal-Heter | 73.9 | -22.2 | 170.0 | 0.114 | ns |
|  |  |  | NP | Gal-Heter | -0.2 | -58.9 | 58.5 | 0.993 | ns |
|  |  | recov | C | Gal-Heter | 114.8 | 92.6 | 137.0 | **0.000** | ******** |
|  |  |  | NP | Gal-Heter | 56.8 | -23.3 | 136.8 | 0.128 | ns |
|  | **Respiration rates (Fig. S1c)** | | | | | | | | |
| **3-way ANOVA** | Effect | | | DFn | DFd | F | p | p<.05 | ges |
|  | Species | | | 1 | 42 | 21.79 | **3.11E-05** | ***** | 0.342 |
|  | Temperature | | | 2 | 42 | 4.07 | **2.40E-02** | ***** | 0.162 |
|  | Condition | | | 1 | 42 | 0.02 | 8.99E-01 |  | 3.86E-04 |
|  | Species:Temperature | | | 2 | 42 | 2.34 | 1.09E-01 |  | 0.100 |
|  | Species:Condition | | | 1 | 42 | 0.01 | 9.22E-01 |  | 2.32E-04 |
|  | Temperature:Condition | | | 2 | 42 | 2.92 | 6.50E-02 |  | 0.122 |
|  | Species:Temperature:Condition | | | 2 | 42 | 3.96 | **2.70E-02** | ***** | 0.159 |
| **Tukey** | Variable | | | Pairwise comparison | estimate | conf.low | conf.high | p.adj | p.adj.signif |
|  | Nutrient condition | Galaxea | ctrl | C-NP | -10.6 | -46.9 | 25.7 | 0.514 | ns |
|  |  |  | heat | C-NP | 6.3 | -29.2 | 41.8 | 0.693 | ns |
|  |  |  | recov | C-NP | 2.8 | -18.0 | 23.5 | 0.747 | ns |
|  |  | Heterox | ctrl | C-NP | 81.3 | -34.3 | 197.0 | 0.144 | ns |
|  |  |  | heat | C-NP | -37.7 | -118.1 | 42.7 | 0.311 | ns |
|  |  |  | recov | C-NP | -63.7 | -105.5 | -21.9 | **0.010** | ****** |
|  | Temperature | Galaxea | C | ctrl-heat | 17.7 | -18.8 | 54.1 | 0.419 | ns |
|  |  |  |  | ctrl-recov | -34.6 | -73.3 | 4.1 | 0.081 | ns |
|  |  |  |  | heat-recov | -52.3 | -90.9 | -13.6 | **0.010** | ****** |
|  |  |  | NP | ctrl-heat | 34.6 | -4.7 | 73.8 | 0.084 | ns |
|  |  |  |  | ctrl-recov | -21.3 | -66.0 | 23.5 | 0.416 | ns |
|  |  |  |  | heat-recov | -55.8 | -98.6 | -13.1 | **0.013** | ***** |
|  |  | Heterox | C | ctrl-heat | 20.5 | -68.8 | 109.7 | 0.813 | ns |
|  |  |  |  | ctrl-recov | 17.1 | -77.5 | 111.8 | 0.878 | ns |
|  |  |  |  | heat-recov | -3.4 | -98.0 | 91.3 | 0.995 | ns |
|  |  |  | NP | ctrl-heat | -98.6 | -211.5 | 14.4 | 0.089 | ns |
|  |  |  |  | ctrl-recov | -127.9 | -247.7 | -8.1 | **0.037** | ***** |
|  |  |  |  | heat-recov | -29.4 | -149.2 | 90.5 | 0.790 | ns |
|  | Species | ctrl | C | Gal-Heter | 56.7 | -32.2 | 145.5 | 0.180 | ns |
|  |  |  | NP | Gal-Heter | 227.9 | 2.9 | 452.9 | **0.048** | ***** |
|  |  | heat | C | Gal-Heter | 73.9 | -22.2 | 170.0 | 0.114 | ns |
|  |  |  | NP | Gal-Heter | -0.2 | -58.9 | 58.5 | 0.993 | ns |
|  |  | recov | C | Gal-Heter | 114.8 | 92.6 | 137.0 | **1.48E-05** | ******** |
|  |  |  | NP | Gal-Heter | 56.8 | -23.3 | 136.8 | 0.128 | ns |
|  | **Carbon tissue content (Fig. S3a)** | | | | | | | | |
| **3-way ANOVA** | Effect | | | DFn | DFd | F | p | p<.05 | ges |
|  | Species | | | 1 | 45 | 0.90 | 3.47E-01 |  | 0.02 |
|  | Temperature | | | 1 | 45 | 1.32 | 2.56E-01 |  | 0.029 |
|  | Condition | | | 2 | 45 | 5.45 | **8.00E-03** | ***** | 0.195 |
|  | Species:Temperature | | | 1 | 45 | 0.08 | 7.82E-01 |  | 0.002 |
|  | Species:Condition | | | 2 | 45 | 14.94 | **1.06E-05** | ***** | 0.399 |
|  | Temperature:Condition | | | 2 | 45 | 6.32 | **4.00E-03** | ***** | 0.219 |
|  | Species:Temperature:Condition | | | 2 | 45 | 3.67 | **3.30E-02** | ***** | 0.14 |
| **Tukey** | Variable | | | Pairwise comparison | estimate | conf.low | conf.high | p.adj | p.adj.signif |
|  | Nutrient condition | Galaxea | ctrl | C-NP | 117.0 | 24.1 | 209.8 | **0.020** | ***** |
|  |  |  | heat | C-NP | -72.9 | -186.6 | 40.8 | 0.178 | ns |
|  |  |  | recov | C-NP | 15.8 | -55.6 | 87.2 | 0.623 | ns |
|  |  | Heterox | ctrl | C-NP | 9.8 | -3.7 | 23.3 | 0.130 | ns |
|  |  |  | heat | C-NP | -17.0 | -66.3 | 32.3 | 0.450 | ns |
|  |  |  | recov | C-NP | 43.1 | 23.3 | 62.8 | **0.002** | ****** |
|  | Temperature | Galaxea | C | ctrl-heat | 170.5 | 47.6 | 293.3 | **0.008** | ****** |
|  |  |  |  | ctrl-recov | -9.5 | -132.4 | 113.4 | 0.977 | ns |
|  |  |  |  | heat-recov | -180.0 | -302.8 | -57.1 | **0.005** | ****** |
|  |  |  | NP | ctrl-heat | -19.4 | -112.6 | 73.8 | 0.845 | ns |
|  |  |  |  | ctrl-recov | -110.7 | -203.9 | -17.5 | **0.021** | ***** |
|  |  |  |  | heat-recov | -91.3 | -184.5 | 1.9 | **0.045** | ***** |
|  |  | Heterox | C | ctrl-heat | 3.7 | -30.5 | 37.9 | 0.953 | ns |
|  |  |  |  | ctrl-recov | 14.8 | -21.3 | 50.8 | 0.522 | ns |
|  |  |  |  | heat-recov | 11.1 | -23.1 | 45.2 | 0.660 | ns |
|  |  |  | NP | ctrl-heat | -23.0 | -66.1 | 20.0 | 0.352 | ns |
|  |  |  |  | ctrl-recov | 48.0 | 2.4 | 93.7 | **0.039** | ***** |
|  |  |  |  | heat-recov | 71.1 | 25.4 | 116.7 | **0.004** | ****** |
|  | Species | ctrl | C | Gal-Heter | 43.5 | -22.3 | 109.3 | 0.162 | ns |
|  |  |  | NP | Gal-Heter | -123.3 | -231.6 | -14.9 | **0.031** | ***** |
|  |  | heat | C | Gal-Heter | 67.8 | 5.3 | 130.3 | **0.037** | ***** |
|  |  |  | NP | Gal-Heter | -63.7 | -138.2 | 10.8 | 0.084 | ns |
|  |  | recov | C | Gal-Heter | -67.3 | -127.5 | -7.2 | **0.033** | ***** |
|  |  |  | NP | Gal-Heter | 95.0 | 37.9 | 152.2 | **0.006** | ****** |
|  | **Nitrogen tissue content (Fig. S3b)** | | | | | | | | |
| **3-way ANOVA** | Effect | | | DFn | DFd | F | p | p<.05 | ges |
|  | Species | | | 1 | 47 | 121.9 | **1.19E-14** | ***** | 0.722 |
|  | Temperature | | | 1 | 47 | 2.0 | 1.62E-01 |  | 0.041 |
|  | Condition | | | 2 | 47 | 27.6 | **1.19E-08** | ***** | 0.54 |
|  | Species:Temperature | | | 1 | 47 | 1.1 | 3.01E-01 |  | 0.023 |
|  | Species:Condition | | | 2 | 47 | 18.6 | **1.09E-06** | ***** | 0.442 |
|  | Temperature:Condition | | | 2 | 47 | 15.0 | **8.93E-06** | ***** | 0.39 |
|  | Species:Temperature:Condition | | | 2 | 47 | 7.7 | **1.00E-03** | ***** | 0.247 |
| **Tukey** | Variable | | | Pairwise comparison | estimate | conf.low | conf.high | p.adj | p.adj.signif |
|  | Nutrient condition | Galaxea | ctrl | C-NP | 25.84 | 6.59 | 45.10 | **0.015** | ***** |
|  |  |  | heat | C-NP | -26.56 | -47.73 | -5.38 | **0.020** | ***** |
|  |  |  | recov | C-NP | -1.88 | -13.39 | 9.62 | 0.710 | ns |
|  |  | Heterox | ctrl | C-NP | -2.24 | -5.71 | 1.22 | 0.174 | ns |
|  |  |  | heat | C-NP | -11.10 | -16.82 | -5.37 | **0.002** | ****** |
|  |  |  | recov | C-NP | -3.54 | -7.21 | 0.14 | 0.057 | ns |
|  | Temperature | Galaxea | C | ctrl-heat | 40.49 | 17.50 | 63.48 | **0.001** | ****** |
|  |  |  |  | ctrl-recov | -8.44 | -31.43 | 14.55 | 0.603 | ns |
|  |  |  |  | heat-recov | -48.93 | -71.93 | -25.94 | **2.80E-04** | ******* |
|  |  |  | NP | ctrl-heat | -11.91 | -29.90 | 6.08 | 0.219 | ns |
|  |  |  |  | ctrl-recov | -36.17 | -55.25 | -17.09 | **0.001** | ******* |
|  |  |  |  | heat-recov | -24.26 | -43.34 | -5.18 | **0.014** | ***** |
|  |  | Heterox | C | ctrl-heat | -5.08 | -9.16 | -1.01 | **0.015** | ***** |
|  |  |  |  | ctrl-recov | -11.87 | -15.95 | -7.80 | **1.40E-05** | ******** |
|  |  |  |  | heat-recov | -6.79 | -10.87 | -2.72 | **0.002** | ****** |
|  |  |  | NP | ctrl-heat | -13.94 | -19.88 | -7.99 | **1.17E-04** | ******* |
|  |  |  |  | ctrl-recov | -13.17 | -19.12 | -7.22 | **1.97E-04** | ******* |
|  |  |  |  | heat-recov | 0.77 | -5.18 | 6.72 | 0.937 | ns |
|  | Species | ctrl | C | Gal-Heter | -6.44 | -18.22 | 5.33 | 0.242 | ns |
|  |  |  | NP | Gal-Heter | -52.02 | -71.76 | -32.28 | **2.97E-04** | ******* |
|  |  | heat | C | Gal-Heter | -9.88 | -18.98 | -0.77 | **0.037** | ***** |
|  |  |  | NP | Gal-Heter | -34.53 | -50.16 | -18.91 | **0.001** | ******* |
|  |  | recov | C | Gal-Heter | -36.56 | -46.13 | -27.00 | **2.16E-05** | ******** |
|  |  |  | NP | Gal-Heter | -11.53 | -17.74 | -5.32 | **0.003** | ****** |
|  | **Phosphorus tissue content (Fig. S3c)** | | | | | | | | |
| **3-way ANOVA** | Effect | | | DFn | DFd | F | p | p<.05 | ges |
|  | Species | | | 1 | 44 | 82.08 | **1.28E-11** | ***** | 0.651 |
|  | Temperature | | | 1 | 44 | 11.33 | **2.00E-03** | ***** | 0.205 |
|  | Condition | | | 2 | 44 | 3.52 | **3.80E-02** | ***** | 0.138 |
|  | Species:Temperature | | | 1 | 44 | 12.73 | **8.84E-04** | ***** | 0.224 |
|  | Species:Condition | | | 2 | 44 | 3.79 | **3.00E-02** | ***** | 0.147 |
|  | Temperature:Condition | | | 2 | 44 | 2.44 | 9.90E-02 |  | 0.1 |
|  | Species:Temperature:Condition | | | 2 | 44 | 0.36 | 7.02E-01 |  | 0.016 |
| **Tukey** | Variable | | | Pairwise comparison | estimate | conf.low | conf.high | p.adj | p.adj.signif |
|  | Nutrient condition | Galaxea | ctrl | C-NP | 1.374 | -1.883 | 4.632 | 0.359 | ns |
|  |  |  | heat | C-NP | 4.321 | 2.321 | 6.321 | **0.001** | ****** |
|  |  |  | recov | C-NP | 3.226 | -0.982 | 7.435 | 0.110 | ns |
|  |  | Heterox | ctrl | C-NP | -0.869 | -2.165 | 0.426 | 0.160 | ns |
|  |  |  | heat | C-NP | 0.430 | -1.225 | 2.084 | 0.566 | ns |
|  |  |  | recov | C-NP | 0.466 | -0.382 | 1.314 | 0.235 | ns |
|  | Temperature | Galaxea | C | ctrl-heat | -3.527 | -8.339 | 1.285 | 0.160 | ns |
|  |  |  |  | ctrl-recov | -0.173 | -4.985 | 4.639 | 0.995 | ns |
|  |  |  |  | heat-recov | 3.354 | -1.718 | 8.427 | 0.215 | ns |
|  |  |  | NP | ctrl-heat | -0.581 | -2.770 | 1.609 | 0.759 | ns |
|  |  |  |  | ctrl-recov | 1.679 | -0.643 | 4.001 | 0.170 | ns |
|  |  |  |  | heat-recov | 2.260 | -0.062 | 4.582 | 0.057 | ns |
|  |  | Heterox | C | ctrl-heat | -0.668 | -2.023 | 0.688 | 0.414 | ns |
|  |  |  |  | ctrl-recov | -0.617 | -1.972 | 0.739 | 0.468 | ns |
|  |  |  |  | heat-recov | 0.051 | -1.304 | 1.407 | 0.994 | ns |
|  |  |  | NP | ctrl-heat | 0.631 | -1.066 | 2.329 | 0.589 | ns |
|  |  |  |  | ctrl-recov | 0.719 | -1.082 | 2.519 | 0.546 | ns |
|  |  |  |  | heat-recov | 0.088 | -1.713 | 1.888 | 0.991 | ns |
|  | Species | ctrl | C | Gal-Heter | -3.089 | -6.035 | -0.142 | **0.042** | ***** |
|  |  |  | NP | Gal-Heter | -0.229 | -2.293 | 1.835 | 0.800 | ns |
|  |  | heat | C | Gal-Heter | -3.532 | -6.909 | -0.156 | **0.043** | ***** |
|  |  |  | NP | Gal-Heter | -5.332 | -7.231 | -3.433 | **1.93E-04** | ******* |
|  |  | recov | C | Gal-Heter | -4.120 | -5.716 | -2.525 | **3.40E-04** | ******* |
|  |  |  | NP | Gal-Heter | -6.293 | -7.992 | -4.593 | **1.01E-04** | ******* |
|  | **C:N ratio (Fig. S3d)** | | | | | | | | |
| **3-way ANOVA** | Effect | | | DFn | DFd | F | p | p<.05 | ges |
|  | Species | | | 1 | 47 | 486.18 | **1.98E-26** | ***** | 0.912 |
|  | Temperature | | | 1 | 47 | 37.28 | **1.86E-07** | ***** | 0.442 |
|  | Condition | | | 2 | 47 | 97.61 | **1.84E-17** | ***** | 0.806 |
|  | Species:Temperature | | | 1 | 47 | 14.08 | **4.80E-04** | ***** | 0.231 |
|  | Species:Condition | | | 2 | 47 | 15.94 | **5.18E-06** | ***** | 0.404 |
|  | Temperature:Condition | | | 2 | 47 | 14.55 | **1.21E-05** | ***** | 0.382 |
|  | Species:Temperature:Condition | | | 2 | 47 | 0.06 | 9.42E-01 |  | 0.003 |
| **Tukey** | Variable | | | Pairwise comparison | estimate | conf.low | conf.high | p.adj | p.adj.signif |
|  | Nutrient condition | Galaxea | ctrl | C-NP | -0.81 | -1.59 | -0.04 | **0.042** | ***** |
|  |  |  | heat | C-NP | 1.16 | 0.40 | 1.92 | **0.008** | ****** |
|  |  |  | recov | C-NP | 0.77 | -0.60 | 2.14 | 0.226 | ns |
|  |  | Heterox | ctrl | C-NP | 0.35 | -0.06 | 0.76 | 0.081 | ns |
|  |  |  | heat | C-NP | 2.25 | 1.47 | 3.04 | **0.0002** | ******* |
|  |  |  | recov | C-NP | 2.13 | 0.96 | 3.30 | **0.003** | ****** |
|  | Temperature | Galaxea | C | ctrl-heat | -1.23 | -2.27 | -0.18 | **0.022** | ***** |
|  |  |  |  | ctrl-recov | 0.93 | -0.11 | 1.98 | 0.081 | ns |
|  |  |  |  | heat-recov | 2.16 | 1.12 | 3.20 | **3.55E-04** | ******* |
|  |  |  | NP | ctrl-heat | 0.75 | -0.42 | 1.91 | 0.236 | ns |
|  |  |  |  | ctrl-recov | 2.52 | 1.28 | 3.75 | **4.91E-04** | ******* |
|  |  |  |  | heat-recov | 1.77 | 0.54 | 3.00 | **0.007** | ****** |
|  |  | Heterox | C | ctrl-heat | 0.75 | -0.04 | 1.54 | 0.062 | ns |
|  |  |  |  | ctrl-recov | 2.72 | 1.94 | 3.51 | **2.39E-06** | ******** |
|  |  |  |  | heat-recov | 1.97 | 1.18 | 2.76 | **6.27E-05** | ******** |
|  |  |  | NP | ctrl-heat | 2.65 | 1.51 | 3.79 | **1.26E-04** | ******* |
|  |  |  |  | ctrl-recov | 4.50 | 3.36 | 5.64 | **5.79E-07** | ******** |
|  |  |  |  | heat-recov | 1.85 | 0.71 | 2.99 | **0.003** | ****** |
|  | Species | ctrl | C | Gal-Heter | 1.70 | 0.95 | 2.45 | **7.76E-04** | ******* |
|  |  |  | NP | Gal-Heter | 3.68 | 3.14 | 4.21 | **2.53E-07** | ******** |
|  |  | heat | C | Gal-Heter | 3.49 | 2.46 | 4.53 | **5.32E-05** | ******** |
|  |  |  | NP | Gal-Heter | 2.87 | 2.41 | 3.33 | **5.48E-07** | ******** |
|  |  | recov | C | Gal-Heter | 4.77 | 3.82 | 5.73 | **2.91E-06** | ******** |
|  |  |  | NP | Gal-Heter | 4.85 | 3.34 | 6.36 | **1.28E-04** | ******* |
|  | **C:P ratio (Fig. S3e)** | | | | | | | | |
| **3-way ANOVA** | Effect | | | DFn | DFd | F | p | p<.05 | ges |
|  | Species | | | 1 | 42 | 46.07 | **2.93E-08** | ***** | 0.523 |
|  | Temperature | | | 1 | 42 | 1.41 | 2.42E-01 |  | 0.032 |
|  | Condition | | | 2 | 42 | 0.86 | 4.29E-01 |  | 0.04 |
|  | Species:Temperature | | | 1 | 42 | 1.20 | 2.79E-01 |  | 0.028 |
|  | Species:Condition | | | 2 | 42 | 2.45 | 9.90E-02 |  | 0.104 |
|  | Temperature:Condition | | | 2 | 42 | 5.89 | **6.00E-03** | ***** | 0.219 |
|  | Species:Temperature:Condition | | | 2 | 42 | 0.18 | 8.36E-01 |  | 0.008 |
| **Tukey** | Variable | | | Pairwise comparison | estimate | conf.low | conf.high | p.adj | p.adj.signif |
|  | Nutrient condition | Galaxea | ctrl | C-NP | 12.14 | -8.98 | 33.27 | 0.216 | ns |
|  |  |  | heat | C-NP | -68.26 | -160.69 | 24.18 | 0.124 | ns |
|  |  |  | recov | C-NP | -26.82 | -60.37 | 6.74 | 0.098 | ns |
|  |  | Heterox | ctrl | C-NP | 56.05 | -14.35 | 126.46 | 0.104 | ns |
|  |  |  | heat | C-NP | -52.36 | -143.20 | 38.47 | 0.215 | ns |
|  |  |  | recov | C-NP | -12.63 | -56.83 | 31.58 | 0.521 | ns |
|  | Temperature | Galaxea | C | ctrl-heat | 82.48 | -24.28 | 189.25 | 0.133 | ns |
|  |  |  |  | ctrl-recov | 16.91 | -89.85 | 123.67 | 0.899 | ns |
|  |  |  |  | heat-recov | -65.57 | -172.33 | 41.19 | 0.252 | ns |
|  |  |  | NP | ctrl-heat | 2.09 | -13.79 | 17.96 | 0.933 | ns |
|  |  |  |  | ctrl-recov | -22.05 | -38.88 | -5.21 | **0.012** | ***** |
|  |  |  |  | heat-recov | -24.13 | -40.97 | -7.30 | **0.007** | ****** |
|  |  | Heterox | C | ctrl-heat | 43.83 | -37.87 | 125.53 | 0.357 | ns |
|  |  |  |  | ctrl-recov | 33.61 | -48.09 | 115.31 | 0.534 | ns |
|  |  |  |  | heat-recov | -10.22 | -91.92 | 71.48 | 0.941 | ns |
|  |  |  | NP | ctrl-heat | -64.59 | -144.54 | 15.36 | 0.117 | ns |
|  |  |  |  | ctrl-recov | -35.07 | -115.02 | 44.87 | 0.478 | ns |
|  |  |  |  | heat-recov | 29.52 | -54.75 | 113.79 | 0.617 | ns |
|  | Species | ctrl | C | Gal-Heter | 72.63 | 50.67 | 94.59 | **1.05E-04** | ******* |
|  |  |  | NP | Gal-Heter | 33.97 | -94.41 | 162.36 | 0.551 | ns |
|  |  | heat | C | Gal-Heter | 89.33 | 38.25 | 140.40 | **4.38E-03** | ****** |
|  |  |  | NP | Gal-Heter | 116.54 | 46.32 | 186.76 | **5.04E-03** | ****** |
|  |  | recov | C | Gal-Heter | 49.87 | 32.17 | 67.57 | **2.87E-04** | ******* |
|  |  |  | NP | Gal-Heter | 103.51 | 88.78 | 118.24 | **2.33E-06** | ******** |
|  | **N:P ratio (Fig. S3f)** | | | | | | | | |
| **3-way ANOVA** | Effect | | | DFn | DFd | F | p | p<.05 | ges |
|  | Species | | | 1 | 47 | 0.69 | 0.412 |  | 0.014 |
|  | Temperature | | | 1 | 47 | 3.89 | 0.055 |  | 0.076 |
|  | Condition | | | 2 | 47 | 3.02 | 0.059 |  | 0.114 |
|  | Species:Temperature | | | 1 | 47 | 2.43 | 0.126 |  | 0.049 |
|  | Species:Condition | | | 2 | 47 | 3.03 | 0.058 |  | 0.114 |
|  | Temperature:Condition | | | 2 | 47 | 5.62 | **0.006** | ***** | 0.193 |
|  | Species:Temperature:Condition | | | 2 | 47 | 1.07 | 0.35 |  | 0.044 |
| **Tukey** | Variable | | | Pairwise comparison | estimate | conf.low | conf.high | p.adj | p.adj.signif |
|  | Nutrient condition | Galaxea | ctrl | C-NP | -0.46 | -8.19 | 7.27 | 0.894 | ns |
|  |  |  | heat | C-NP | -20.22 | -41.68 | 1.25 | 0.062 | ns |
|  |  |  | recov | C-NP | -0.45 | -10.48 | 9.57 | 0.918 | ns |
|  |  | Heterox | ctrl | C-NP | 5.36 | -1.70 | 12.41 | 0.118 | ns |
|  |  |  | heat | C-NP | -6.49 | -15.90 | 2.92 | 0.150 | ns |
|  |  |  | recov | C-NP | -1.39 | -6.60 | 3.83 | 0.557 | ns |
|  | Temperature | Galaxea | C | ctrl-heat | 19.01 | -3.83 | 41.85 | 0.107 | ns |
|  |  |  |  | ctrl-recov | -2.07 | -26.30 | 22.16 | 0.971 | ns |
|  |  |  |  | heat-recov | -21.08 | -45.30 | 3.15 | 0.090 | ns |
|  |  |  | NP | ctrl-heat | -0.75 | -8.75 | 7.26 | 0.967 | ns |
|  |  |  |  | ctrl-recov | -2.06 | -10.07 | 5.94 | 0.775 | ns |
|  |  |  |  | heat-recov | -1.32 | -9.32 | 6.69 | 0.900 | ns |
|  |  | Heterox | C | ctrl-heat | 3.66 | -5.29 | 12.61 | 0.537 | ns |
|  |  |  |  | ctrl-recov | -0.45 | -9.40 | 8.50 | 0.990 | ns |
|  |  |  |  | heat-recov | -4.11 | -13.06 | 4.84 | 0.462 | ns |
|  |  |  | NP | ctrl-heat | -8.19 | -16.42 | 0.04 | 0.051 | ns |
|  |  |  |  | ctrl-recov | -7.19 | -15.42 | 1.04 | 0.089 | ns |
|  |  |  |  | heat-recov | 1.00 | -7.23 | 9.22 | 0.944 | ns |
|  | Species | ctrl | C | Gal-Heter | 3.22 | -4.29 | 10.73 | 0.352 | ns |
|  |  |  | NP | Gal-Heter | -12.13 | -35.23 | 10.97 | 0.260 | ns |
|  |  | heat | C | Gal-Heter | 4.84 | -0.70 | 10.37 | 0.078 | ns |
|  |  |  | NP | Gal-Heter | 9.03 | 1.74 | 16.33 | **0.021** | ***** |
|  |  | recov | C | Gal-Heter | 1.59 | -2.36 | 5.55 | 0.380 | ns |
|  |  |  | NP | Gal-Heter | 3.90 | -4.98 | 12.79 | 0.340 | ns |
|  | **Protein content (Fig. 4)** | | | | | | | | |
| **3-way ANOVA** | Effect | | | DFn | DFd | F | p | p<.05 | ges |
|  | Species | | | 1 | 46 | 0.80 | 0.375 |  | 0.017 |
|  | Temperature | | | 1 | 46 | 0.42 | 0.519 |  | 0.009 |
|  | Condition | | | 2 | 46 | 0.46 | 0.637 |  | 0.019 |
|  | Species:Temperature | | | 1 | 46 | 3.37 | 0.073 |  | 0.068 |
|  | Species:Condition | | | 2 | 46 | 0.51 | 0.605 |  | 0.022 |
|  | Temperature:Condition | | | 2 | 46 | 5.79 | **0.006** | ***** | 0.201 |
|  | Species:Temperature:Condition | | | 2 | 46 | 0.86 | 0.431 |  | 0.036 |
| **Tukey** | Variable | | | Pairwise comparison | estimate | conf.low | conf.high | p.adj | p.adj.signif |
|  | Nutrient condition | Galaxea | ctrl | C-NP | 72.59 | -3.94 | 149.13 | 0.060 | ns |
|  |  |  | heat | C-NP | -23.94 | -79.76 | 31.87 | 0.344 | ns |
|  |  |  | recov | C-NP | -18.05 | -90.39 | 54.29 | 0.581 | ns |
|  |  | Heterox | ctrl | C-NP | 3.90 | -30.96 | 38.76 | 0.803 | ns |
|  |  |  | heat | C-NP | -52.93 | -86.14 | -19.73 | **0.007** | ****** |
|  |  |  | recov | C-NP | -26.55 | -61.83 | 8.73 | 0.121 | ns |
|  | Temperature | Galaxea | C | ctrl-heat | 49.52 | -49.51 | 148.55 | 0.404 | ns |
|  |  |  |  | ctrl-recov | 53.00 | -46.03 | 152.03 | 0.358 | ns |
|  |  |  |  | heat-recov | 3.48 | -95.55 | 102.51 | 0.995 | ns |
|  |  |  | NP | ctrl-heat | -47.01 | -98.96 | 4.93 | 0.077 | ns |
|  |  |  |  | ctrl-recov | -37.64 | -86.62 | 11.33 | 0.140 | ns |
|  |  |  |  | heat-recov | 9.37 | -42.58 | 61.32 | 0.879 | ns |
|  |  | Heterox | C | ctrl-heat | 5.82 | -28.59 | 40.24 | 0.895 | ns |
|  |  |  |  | ctrl-recov | 11.46 | -22.95 | 45.87 | 0.657 | ns |
|  |  |  |  | heat-recov | 5.64 | -28.78 | 40.05 | 0.901 | ns |
|  |  |  | NP | ctrl-heat | -51.01 | -97.78 | -4.25 | **0.033** | ***** |
|  |  |  |  | ctrl-recov | -18.99 | -63.08 | 25.10 | 0.498 | ns |
|  |  |  |  | heat-recov | 32.02 | -14.75 | 78.78 | 0.199 | ns |
|  | Species | ctrl | C | Gal-Heter | 36.51 | -31.18 | 104.19 | 0.249 | ns |
|  |  |  | NP | Gal-Heter | -7.19 | -57.12 | 42.74 | 0.748 | ns |
|  |  | heat | C | Gal-Heter | -5.03 | -77.44 | 67.38 | 0.877 | ns |
|  |  |  | NP | Gal-Heter | -32.18 | -82.09 | 17.73 | 0.175 | ns |
|  |  | recov | C | Gal-Heter | -36.18 | -70.48 | -1.88 | **0.042** | ***** |
|  |  |  | NP | Gal-Heter | -13.53 | -48.66 | 21.61 | 0.400 | ns |
|  | **Lipid content (Fig. 4)** | | | | | | | | |
| **3-way ANOVA** | Effect | | | DFn | DFd | F | p | p<.05 | ges |
|  | Species | | | 1 | 47 | 440.61 | **1.62E-25** | ***** | 0.904 |
|  | Temperature | | | 1 | 47 | 26.10 | **5.82E-06** | ***** | 0.357 |
|  | Condition | | | 2 | 47 | 86.93 | **1.61E-16** | ***** | 0.787 |
|  | Species:Temperature | | | 1 | 47 | 11.45 | **1.00E-03** | ***** | 0.196 |
|  | Species:Condition | | | 2 | 47 | 35.18 | **4.57E-10** | ***** | 0.6 |
|  | Temperature:Condition | | | 2 | 47 | 7.05 | **2.00E-03** | ***** | 0.231 |
|  | Species:Temperature:Condition | | | 2 | 47 | 4.71 | **1.40E-02** | ***** | 0.167 |
| **Tukey** | Variable | | | Pairwise comparison | estimate | conf.low | conf.high | p.adj | p.adj.signif |
|  | Nutrient condition | Galaxea | ctrl | C-NP | 23.7 | 2.1 | 45.3 | **0.035** | ***** |
|  |  |  | heat | C-NP | -5.2 | -30.1 | 19.7 | 0.644 | ns |
|  |  |  | recov | C-NP | 21.5 | -32.4 | 75.3 | 0.385 | ns |
|  |  | Heterox | ctrl | C-NP | 25.9 | 8.8 | 43.1 | **0.009** | ****** |
|  |  |  | heat | C-NP | 30.3 | -1.6 | 62.3 | 0.060 | ns |
|  |  |  | recov | C-NP | 135.0 | 61.7 | 208.4 | **0.003** | ****** |
|  | Temperature | Galaxea | C | ctrl-heat | 20.7 | -9.1 | 50.6 | 0.195 | ns |
|  |  |  |  | ctrl-recov | 43.5 | 13.6 | 73.4 | **0.006** | ****** |
|  |  |  |  | heat-recov | 22.7 | -7.2 | 52.6 | 0.147 | ns |
|  |  |  | NP | ctrl-heat | -8.1 | -59.7 | 43.4 | 0.908 | ns |
|  |  |  |  | ctrl-recov | 41.3 | -10.3 | 92.8 | 0.124 | ns |
|  |  |  |  | heat-recov | 49.4 | -2.2 | 101.0 | 0.061 | ns |
|  |  | Heterox | C | ctrl-heat | 25.7 | -33.6 | 85.1 | 0.500 | ns |
|  |  |  |  | ctrl-recov | 133.0 | 73.6 | 192.4 | **1.76E-04** | ******* |
|  |  |  |  | heat-recov | 107.3 | 47.9 | 166.6 | **0.001** | ****** |
|  |  |  | NP | ctrl-heat | 30.2 | -24.6 | 84.9 | 0.333 | ns |
|  |  |  |  | ctrl-recov | 242.1 | 187.4 | 296.9 | **3.36E-07** | ******** |
|  |  |  |  | heat-recov | 212.0 | 160.4 | 263.6 | **7.17E-07** | ******** |
|  | Species | ctrl | C | Gal-Heter | 101.6 | 77.5 | 125.7 | **1.04E-05** | ******** |
|  |  |  | NP | Gal-Heter | 106.6 | 75.3 | 137.8 | **4.92E-05** | ******** |
|  |  | heat | C | Gal-Heter | 191.1 | 132.8 | 249.4 | **6.53E-05** | ******** |
|  |  |  | NP | Gal-Heter | 103.8 | 92.0 | 115.7 | **1.23E-07** | ******** |
|  |  | recov | C | Gal-Heter | 142.1 | 116.4 | 167.9 | **1.37E-06** | ******** |
|  |  |  | NP | Gal-Heter | 304.7 | 234.8 | 374.6 | **8.16E-06** | ******** |
|  | **Carbohydrate content (Fig. 4)** | | | | | | | | |
| **3-way ANOVA** | Effect | | | DFn | DFd | F | p | p<.05 | ges |
|  | Species | | | 1 | 45 | 7.66 | **8.00E-03** | ***** | 0.145 |
|  | Temperature | | | 1 | 45 | 0.50 | 4.85E-01 |  | 0.011 |
|  | Condition | | | 2 | 45 | 50.19 | **3.47E-12** | ***** | 0.69 |
|  | Species:Temperature | | | 1 | 45 | 0.19 | 6.62E-01 |  | 0.004 |
|  | Species:Condition | | | 2 | 45 | 2.56 | 8.90E-02 |  | 0.102 |
|  | Temperature:Condition | | | 2 | 45 | 5.07 | **1.00E-02** | ***** | 0.184 |
|  | Species:Temperature:Condition | | | 2 | 45 | 11.45 | **9.53E-05** | ***** | 0.337 |
| **Tukey** | Variable | | | Pairwise comparison | estimate | conf.low | conf.high | p.adj | p.adj.signif |
|  | Nutrient condition | Galaxea | ctrl | C-NP | 5.0 | -29.9 | 39.9 | 0.750 | ns |
|  |  |  | heat | C-NP | 50.6 | 26.3 | 75.0 | **0.002** | ****** |
|  |  |  | recov | C-NP | -81.7 | -146.1 | -17.2 | **0.020** | ***** |
|  |  | Heterox | ctrl | C-NP | -4.7 | -48.3 | 38.9 | 0.811 | ns |
|  |  |  | heat | C-NP | -11.0 | -30.3 | 8.3 | 0.225 | ns |
|  |  |  | recov | C-NP | 13.2 | -14.1 | 40.4 | 0.292 | ns |
|  | Temperature | Galaxea | C | ctrl-heat | -12.7 | -63.2 | 37.9 | 0.786 | ns |
|  |  |  |  | ctrl-recov | 117.8 | 67.2 | 168.3 | **1.24E-04** | ******* |
|  |  |  |  | heat-recov | 130.4 | 79.8 | 181.0 | **4.68E-05** | ******** |
|  |  |  | NP | ctrl-heat | 33.0 | -15.7 | 81.7 | 0.201 | ns |
|  |  |  |  | ctrl-recov | 31.1 | -17.6 | 79.8 | 0.235 | ns |
|  |  |  |  | heat-recov | -1.9 | -53.2 | 49.4 | 0.994 | ns |
|  |  | Heterox | C | ctrl-heat | -24.8 | -68.4 | 18.9 | 0.314 | ns |
|  |  |  |  | ctrl-recov | 43.4 | -2.9 | 89.7 | 0.067 | ns |
|  |  |  |  | heat-recov | 68.2 | 21.8 | 114.5 | **0.006** | ****** |
|  |  |  | NP | ctrl-heat | -31.1 | -59.6 | -2.6 | **0.033** | ***** |
|  |  |  |  | ctrl-recov | 61.2 | 32.7 | 89.7 | **2.57E-04** | ******* |
|  |  |  |  | heat-recov | 92.3 | 63.8 | 120.8 | **4.70E-06** | ******** |
|  | Species | ctrl | C | Gal-Heter | 42.3 | -1.2 | 85.8 | 0.055 | ns |
|  |  |  | NP | Gal-Heter | 30.2 | 6.6 | 53.9 | **0.019** | ***** |
|  |  | heat | C | Gal-Heter | -32.0 | -88.1 | 24.0 | 0.219 | ns |
|  |  |  | NP | Gal-Heter | 32.7 | -2.3 | 67.7 | 0.064 | ns |
|  |  | recov | C | Gal-Heter | -31.4 | -49.8 | -13.0 | **0.005** | ****** |
|  |  |  | NP | Gal-Heter | 62.8 | 20.9 | 104.7 | **0.009** | ****** |
|  | **Total tissue energy content (Fig. 4d)** | | | | | | | | |
| **3-way ANOVA** | Effect | | | DFn | DFd | F | p | p<.05 | ges |
|  | Species | | | 1 | 48 | 215.24 | **2.30E-19** | ***** | 0.818 |
|  | Temperature | | | 1 | 48 | 8.85 | **5.00E-03** | ***** | 0.156 |
|  | Condition | | | 2 | 48 | 75.70 | **1.43E-15** | ***** | 0.759 |
|  | Species:Temperature | | | 1 | 48 | 3.72 | 6.00E-02 |  | 0.072 |
|  | Species:Condition | | | 2 | 48 | 16.56 | **3.40E-06** | ***** | 0.408 |
|  | Temperature:Condition | | | 2 | 48 | 0.68 | 5.11E-01 |  | 0.028 |
|  | Species:Temperature:Condition | | | 2 | 48 | 10.44 | **1.72E-04** | ***** | 0.303 |
| **Tukey** | Variable | | | Pairwise comparison | estimate | conf.low | conf.high | p.adj | p.adj.signif |
|  | Nutrient condition | Galaxea | ctrl | C-NP | 2.33 | -0.02 | 4.67 | 0.051 | ns |
|  |  |  | heat | C-NP | 0.89 | -1.13 | 2.92 | 0.339 | ns |
|  |  |  | recov | C-NP | -1.84 | -5.28 | 1.61 | 0.254 | ns |
|  |  | Heterox | ctrl | C-NP | 0.63 | -0.57 | 1.83 | 0.261 | ns |
|  |  |  | heat | C-NP | 0.33 | -1.32 | 1.98 | 0.654 | ns |
|  |  |  | recov | C-NP | 5.51 | 2.12 | 8.90 | **0.006** | ****** |
|  | Temperature | Galaxea | C | ctrl-heat | 1.38 | -2.02 | 4.79 | 0.541 | ns |
|  |  |  |  | ctrl-recov | 5.46 | 2.06 | 8.86 | **0.003** | ****** |
|  |  |  |  | heat-recov | 4.08 | 0.67 | 7.48 | **0.020** | ***** |
|  |  |  | NP | ctrl-heat | -0.05 | -2.80 | 2.70 | 0.999 | ns |
|  |  |  |  | ctrl-recov | 1.30 | -1.45 | 4.05 | 0.444 | ns |
|  |  |  |  | heat-recov | 1.34 | -1.41 | 4.09 | 0.419 | ns |
|  |  | Heterox | C | ctrl-heat | 0.53 | -2.15 | 3.21 | 0.861 | ns |
|  |  |  |  | ctrl-recov | 6.16 | 3.48 | 8.84 | **1.40E-04** | ******* |
|  |  |  |  | heat-recov | 5.63 | 2.95 | 8.31 | **3.13E-04** | ******* |
|  |  |  | NP | ctrl-heat | 0.23 | -2.37 | 2.84 | 0.970 | ns |
|  |  |  |  | ctrl-recov | 11.05 | 8.44 | 13.65 | **2.61E-07** | ******** |
|  |  |  |  | heat-recov | 10.81 | 8.21 | 13.42 | **3.29E-07** | ******** |
|  | Species | ctrl | C | Gal-Heter | 5.66 | 3.27 | 8.06 | **6.12E-04** | ******* |
|  |  |  | NP | Gal-Heter | 4.81 | 2.88 | 6.74 | **4.31E-04** | ******* |
|  |  | heat | C | Gal-Heter | 6.36 | 2.96 | 9.77 | **0.003** | ****** |
|  |  |  | NP | Gal-Heter | 3.97 | 2.88 | 5.05 | **2.99E-05** | ******** |
|  |  | recov | C | Gal-Heter | 4.24 | 2.48 | 6.01 | **5.45E-04** | ******* |
|  |  |  | NP | Gal-Heter | 13.72 | 10.28 | 17.15 | **1.56E-05** | ******** |

**Table S2.** **Statistical analysis and results of the effects of nutrient enrichment and warming on coral carbon budget.** “C”, control, non-enriched nubbins; “NP”, nutrient-enriched nubbins; “*ctrl*”, control temperature at 25°C; “heat”, short-term heat stress at 30°C for 14 days; “recov”, recovery phase, with temperature going back to 25°C for 14 days. Significant differences are displayed in bold.

|  | **P_C_ (Fig. S2a)** | | | | | | | | |
| --- | --- | --- | --- | --- | --- | --- | --- | --- | --- |
| **3-way ANOVA** | Effect | | | DFn | DFd | F | p | p<.05 | ges |
|  | Species | | | 1 | 43 | 20.42 | **4.80E-05** | ***** | 0.322 |
|  | Temperature | | | 2 | 43 | 6.78 | **0.003** | ***** | 0.24 |
|  | Condition | | | 1 | 43 | 1.38 | 0.247 |  | 0.031 |
|  | Species:Temperature | | | 2 | 43 | 2.58 | 0.088 |  | 0.107 |
|  | Species:Condition | | | 1 | 43 | 0.24 | 0.630 |  | 0.005 |
|  | Temperature:Condition | | | 2 | 43 | 1.38 | 0.262 |  | 0.06 |
|  | Species:Temperature:Condition | | | 2 | 43 | 4.21 | **0.021** | ***** | 0.164 |
| **Tukey** | Variable | | | Pairwise comparison | estimate | conf.low | conf.high | p.adj | p.adj.signif |
|  | Nutrient condition | Galaxea | ctrl | C-NP | -356.0 | -967.4 | 255.3 | 0.216 | ns |
|  |  |  | heat | C-NP | 755.2 | 227.9 | 1282.4 | **0.011** | ***** |
|  |  |  | recov | C-NP | 33.6 | -274.0 | 341.3 | 0.790 | ns |
|  |  | Heterox | ctrl | C-NP | 1490.7 | -751.3 | 3732.7 | 0.164 | ns |
|  |  |  | heat | C-NP | -53.4 | -1163.0 | 1056.2 | 0.914 | ns |
|  |  |  | recov | C-NP | -599.7 | -1300.4 | 101.0 | **0.041** | ***** |
|  | Temperature | Galaxea | C | ctrl-heat | -112.1 | -729.3 | 505.0 | 0.877 | ns |
|  |  |  |  | ctrl-recov | -836.2 | -1490.8 | -181.6 | **0.014** | ***** |
|  |  |  |  | heat-recov | -724.0 | -1378.6 | -69.5 | **0.031** | ***** |
|  |  |  | NP | ctrl-heat | 999.0 | 414.1 | 1584.0 | **0.002** | ****** |
|  |  |  |  | ctrl-recov | -446.5 | -1121.9 | 228.9 | 0.215 | ns |
|  |  |  |  | heat-recov | -1445.6 | -2121.0 | -770.1 | **0.000** | ******* |
|  |  | Heterox | C | ctrl-heat | 75.5 | -1222.3 | 1373.4 | 0.986 | ns |
|  |  |  |  | ctrl-recov | -201.8 | -1578.4 | 1174.7 | 0.918 | ns |
|  |  |  |  | heat-recov | -277.4 | -1654.0 | 1099.2 | 0.851 | ns |
|  |  |  | NP | ctrl-heat | -1468.6 | -3663.9 | 726.8 | 0.212 | ns |
|  |  |  |  | ctrl-recov | -2292.2 | -4620.8 | 36.3 | **0.044** | ***** |
|  |  |  |  | heat-recov | -823.7 | -3152.2 | 1504.9 | 0.618 | ns |
|  | Species | ctrl | C | Gal-Heter | 618.4 | -351.1 | 1587.8 | 0.180 | ns |
|  |  |  | NP | Gal-Heter | 2465.1 | 353.1 | 4577.0 | **0.027** | ***** |
|  |  | heat | C | Gal-Heter | 806.1 | -242.3 | 1854.4 | 0.114 | ns |
|  |  |  | NP | Gal-Heter | -2.5 | -643.0 | 638.0 | 0.993 | ns |
|  |  | recov | C | Gal-Heter | 1252.7 | 1010.4 | 1495.0 | **0.000** | ******** |
|  |  |  | NP | Gal-Heter | 619.4 | -253.8 | 1492.6 | 0.128 | ns |
|  | **Symbiont incorporation rate (Fig. S2b)** | | | | | | | | |
| **3-way ANOVA** | Effect | | | DFn | DFd | F | p | p<.05 | ges |
|  | Species | | | 1 | 36 | 39.18 | **3.13E-07** | ***** | 0.521 |
|  | Temperature | | | 2 | 36 | 6.04 | **5.00E-03** | ***** | 0.251 |
|  | Condition | | | 1 | 36 | 0.01 | 9.06E-01 |  | 0.000391 |
|  | Species:Temperature | | | 2 | 36 | 1.68 | 2.01E-01 |  | 0.085 |
|  | Species:Condition | | | 1 | 36 | 1.09 | 3.03E-01 |  | 0.029 |
|  | Temperature:Condition | | | 2 | 36 | 3.29 | **4.90E-02** | ***** | 0.155 |
|  | Species:Temperature:Condition | | | 2 | 36 | 7.22 | **2.00E-03** | ***** | 0.286 |
| **Tukey** | Variable | | | Pairwise comparison | estimate | conf.low | conf.high | p.adj | p.adj.signif |
|  | Nutrient condition | Galaxea | ctrl | C-NP | -4.00 | -12.83 | 4.84 | 0.311 | ns |
|  |  |  | heat | C-NP | -0.46 | -2.82 | 1.90 | 0.653 | ns |
|  |  |  | recov | C-NP | 0.30 | -2.63 | 3.23 | 0.813 | ns |
|  |  | Heterox | ctrl | C-NP | 15.19 | 4.21 | 26.17 | **0.015** | ***** |
|  |  |  | heat | C-NP | -4.97 | -14.69 | 4.75 | 0.257 | ns |
|  |  |  | recov | C-NP | -5.00 | -18.21 | 8.21 | 0.390 | ns |
|  | Temperature | Galaxea | C | ctrl-heat | -4.37 | -12.77 | 4.03 | 0.357 | ns |
|  |  |  |  | ctrl-recov | -5.32 | -13.73 | 3.08 | 0.234 | ns |
|  |  |  |  | heat-recov | -0.95 | -9.36 | 7.45 | 0.946 | ns |
|  |  |  | NP | ctrl-heat | -0.83 | -3.91 | 2.25 | 0.740 | ns |
|  |  |  |  | ctrl-recov | -1.03 | -4.11 | 2.04 | 0.632 | ns |
|  |  |  |  | heat-recov | -0.20 | -3.28 | 2.87 | 0.982 | ns |
|  |  | Heterox | C | ctrl-heat | 6.12 | -8.68 | 20.92 | 0.507 | ns |
|  |  |  |  | ctrl-recov | 0.54 | -14.26 | 15.35 | 0.994 | ns |
|  |  |  |  | heat-recov | -5.58 | -20.38 | 9.23 | 0.565 | ns |
|  |  |  | NP | ctrl-heat | -14.04 | -24.95 | -3.13 | **0.015** | ***** |
|  |  |  |  | ctrl-recov | -19.65 | -30.56 | -8.74 | **0.002** | ****** |
|  |  |  |  | heat-recov | -5.61 | -16.52 | 5.30 | 0.365 | ns |
|  | Species | ctrl | C | Gal-Heter | 2.34 | -7.85 | 12.53 | 0.594 | ns |
|  |  |  | NP | Gal-Heter | 21.53 | 11.79 | 31.27 | **0.002** | ****** |
|  |  | heat | C | Gal-Heter | 12.83 | 3.41 | 22.26 | **0.016** | ***** |
|  |  |  | NP | Gal-Heter | 8.32 | 4.96 | 11.68 | **0.001** | ******* |
|  |  | recov | C | Gal-Heter | 8.21 | -3.67 | 20.10 | 0.142 | ns |
|  |  |  | NP | Gal-Heter | 2.92 | -3.55 | 9.38 | 0.312 | ns |
|  | **Symbiont respiration per cell (Fig. S2c)** | | | | | | | | |
| **3-way ANOVA** | Effect | | | DFn | DFd | F | p | p<.05 | ges |
|  | Species | | | 1 | 32 | 25.32 | **1.81E-05** | ***** | 0.442 |
|  | Temperature | | | 2 | 32 | 8.43 | **1.00E-03** | ***** | 0.345 |
|  | Condition | | | 1 | 32 | 0.87 | 3.59E-01 |  | 0.026 |
|  | Species:Temperature | | | 2 | 32 | 3.59 | **3.90E-02** | ***** | 0.183 |
|  | Species:Condition | | | 1 | 32 | 0.24 | 6.29E-01 |  | 0.007 |
|  | Temperature:Condition | | | 2 | 32 | 17.12 | **8.78E-06** | ***** | 0.517 |
|  | Species:Temperature:Condition | | | 2 | 32 | 13.29 | **6.27E-05** | ***** | 0.454 |
| **Tukey** | Variable | | | Pairwise comparison | estimate | conf.low | conf.high | p.adj | p.adj.signif |
|  | Nutrient condition | Galaxea | ctrl | C-NP | 13.99 | 2.37 | 25.62 | **0.027** | ***** |
|  |  |  | heat | C-NP | -0.53 | -2.06 | 1.00 | 0.429 | ns |
|  |  |  | recov | C-NP | -8.26 | -13.91 | -2.60 | **0.012** | ***** |
|  |  | Heterox | ctrl | C-NP | 1.34 | 0.37 | 2.32 | **0.017** | ***** |
|  |  |  | heat | C-NP | -0.17 | -1.00 | 0.66 | 0.599 | ns |
|  |  |  | recov | C-NP | 0.04 | -0.21 | 0.30 | 0.687 | ns |
|  | Temperature | Galaxea | C | ctrl-heat | 0.90 | -5.36 | 7.16 | 0.912 | ns |
|  |  |  |  | ctrl-recov | 8.33 | 2.08 | 14.59 | **0.013** | ***** |
|  |  |  |  | heat-recov | 7.43 | 1.64 | 13.23 | **0.016** | ***** |
|  |  |  | NP | ctrl-heat | -13.62 | -22.41 | -4.84 | **0.005** | ****** |
|  |  |  |  | ctrl-recov | -13.92 | -22.70 | -5.13 | **0.004** | ****** |
|  |  |  |  | heat-recov | -0.29 | -9.08 | 8.49 | 0.995 | ns |
|  |  | Heterox | C | ctrl-heat | -0.13 | -0.73 | 0.47 | 0.815 | ns |
|  |  |  |  | ctrl-recov | -0.62 | -1.18 | -0.07 | **0.031** | ***** |
|  |  |  |  | heat-recov | -0.49 | -1.10 | 0.11 | 0.107 | ns |
|  |  |  | NP | ctrl-heat | -1.64 | -2.67 | -0.61 | **0.006** | ****** |
|  |  |  |  | ctrl-recov | -1.92 | -2.89 | -0.96 | **0.002** | ****** |
|  |  |  |  | heat-recov | -0.28 | -1.25 | 0.69 | 0.685 | ns |
|  | Species | ctrl | C | Gal-Heter | -0.20 | -0.86 | 0.47 | 0.480 | ns |
|  |  |  | NP | Gal-Heter | -12.85 | -24.49 | -1.20 | **0.036** | ***** |
|  |  | heat | C | Gal-Heter | -1.23 | -2.49 | 0.04 | 0.055 | ns |
|  |  |  | NP | Gal-Heter | -0.87 | -2.43 | 0.70 | 0.214 | ns |
|  |  | recov | C | Gal-Heter | -9.16 | -14.80 | -3.52 | **0.007** | ****** |
|  |  |  | NP | Gal-Heter | -0.85 | -1.33 | -0.37 | **0.005** | ****** |
|  | **Host incorporation rate (Fig. S2d)** | | | | | | | | |
| **3-way ANOVA** | Effect | | | DFn | DFd | F | p | p<.05 | ges |
|  | Species | | | 1 | 35 | 2.11 | 0.155 |  | 0.057 |
|  | Temperature | | | 2 | 35 | 2.28 | 0.118 |  | 0.115 |
|  | Condition | | | 1 | 35 | 0.11 | 0.74 |  | 0.003 |
|  | Species:Temperature | | | 2 | 35 | 1.71 | 0.197 |  | 0.089 |
|  | Species:Condition | | | 1 | 35 | 4.98 | **0.032** | ***** | 0.125 |
|  | Temperature:Condition | | | 2 | 35 | 1.64 | 0.208 |  | 0.086 |
|  | Species:Temperature:Condition | | | 2 | 35 | 2.28 | 0.118 |  | 0.115 |
| **Tukey** | Variable | | | Pairwise comparison | estimate | conf.low | conf.high | p.adj | p.adj.signif |
|  | Nutrient condition | Galaxea | ctrl | C-NP | 0.79 | -2.18 | 3.76 | 0.539 | ns |
|  |  |  | heat | C-NP | 1.11 | -1.42 | 3.65 | 0.324 | ns |
|  |  |  | recov | C-NP | 1.19 | 0.25 | 2.12 | **0.021** | ***** |
|  |  | Heterox | ctrl | C-NP | 1.40 | -2.36 | 5.16 | 0.383 | ns |
|  |  |  | heat | C-NP | -4.00 | -9.14 | 1.14 | 0.106 | ns |
|  |  |  | recov | C-NP | -1.36 | -4.30 | 1.58 | 0.302 | ns |
|  | Temperature | Galaxea | C | ctrl-heat | 0.54 | -1.49 | 2.58 | 0.743 | ns |
|  |  |  |  | ctrl-recov | 0.33 | -1.70 | 2.36 | 0.894 | ns |
|  |  |  |  | heat-recov | -0.21 | -2.24 | 1.82 | 0.954 | ns |
|  |  |  | NP | ctrl-heat | 0.87 | -2.27 | 4.01 | 0.729 | ns |
|  |  |  |  | ctrl-recov | 0.73 | -2.41 | 3.87 | 0.798 | ns |
|  |  |  |  | heat-recov | -0.14 | -3.28 | 3.00 | 0.992 | ns |
|  |  | Heterox | C | ctrl-heat | 4.17 | -2.15 | 10.49 | 0.204 | ns |
|  |  |  |  | ctrl-recov | 0.32 | -6.00 | 6.64 | 0.988 | ns |
|  |  |  |  | heat-recov | -3.85 | -9.70 | 2.00 | 0.206 | ns |
|  |  |  | NP | ctrl-heat | -1.23 | -4.28 | 1.83 | 0.526 | ns |
|  |  |  |  | ctrl-recov | -2.44 | -5.49 | 0.62 | 0.12 | ns |
|  |  |  |  | heat-recov | -1.21 | -4.27 | 1.84 | 0.534 | ns |
|  | Species | ctrl | C | Gal-Heter | 0.76 | 0.10 | 1.42 | **0.032** | ***** |
|  |  |  | NP | Gal-Heter | 1.37 | -2.84 | 5.57 | 0.457 | ns |
|  |  | heat | C | Gal-Heter | 4.38 | -1.05 | 9.82 | 0.096 | ns |
|  |  |  | NP | Gal-Heter | -0.73 | -2.55 | 1.09 | 0.367 | ns |
|  |  | recov | C | Gal-Heter | 0.75 | -2.15 | 3.65 | 0.552 | ns |
|  |  |  | NP | Gal-Heter | -1.80 | -2.85 | -0.75 | **0.006** | ****** |
|  | **Translocation rate per AFDW (Fig. S2e)** | | | | | | | | |
| **3-way ANOVA** | Effect | | | DFn | DFd | F | p | p<.05 | ges |
|  | Species | | | 1 | 35 | 1244.63 | **6.02E-29** | ***** | 0.973 |
|  | Temperature | | | 2 | 35 | 558.66 | **2.78E-27** | ***** | 0.97 |
|  | Condition | | | 1 | 35 | 135.05 | **1.45E-13** | ***** | 0.794 |
|  | Species:Temperature | | | 2 | 35 | 310.95 | **5.19E-23** | ***** | 0.947 |
|  | Species:Condition | | | 1 | 35 | 118.21 | **9.08E-13** | ***** | 0.772 |
|  | Temperature:Condition | | | 2 | 35 | 114.98 | **4.13E-16** | ***** | 0.868 |
|  | Species:Temperature:Condition | | | 2 | 35 | 678.49 | **1.02E-28** | ***** | 0.975 |
| **Tukey** | Variable | | | Pairwise comparison | estimate | conf.low | conf.high | p.adj | p.adj.signif |
|  | Nutrient condition | Galaxea | ctrl | C-NP | -929.7 | -1035.7 | -823.6 | **7.1E-07** | ******** |
|  |  |  | heat | C-NP | 736.6 | 708.3 | 764.9 | **1.8E-11** | ******** |
|  |  |  | recov | C-NP | 244.8 | 209.7 | 280.0 | **2.5E-06** | ******** |
|  |  | Heterox | ctrl | C-NP | 1429.4 | 1273.9 | 1584.9 | **3.9E-06** | ******** |
|  |  |  | heat | C-NP | 398.6 | 282.0 | 515.3 | **1.6E-04** | ******* |
|  |  |  | recov | C-NP | -437.5 | -596.1 | -279.0 | **5.1E-04** | ******* |
|  | Temperature | Galaxea | C | ctrl-heat | -84.7 | -185.5 | 16.2 | 1.0E-01 | ns |
|  |  |  |  | ctrl-recov | -963.7 | -1064.5 | -862.9 | **2.1E-09** | ******** |
|  |  |  |  | heat-recov | -879.0 | -979.9 | -778.2 | **3.6E-09** | ******** |
|  |  |  | NP | ctrl-heat | 1581.6 | 1544.7 | 1618.5 | **5.5E-10** | ******** |
|  |  |  |  | ctrl-recov | 210.8 | 173.9 | 247.8 | **1.9E-07** | ******** |
|  |  |  |  | heat-recov | -1370.8 | -1407.7 | -1333.8 | **5.5E-10** | ******** |
|  |  | Heterox | C | ctrl-heat | 274.5 | 71.2 | 477.9 | **1.2E-02** | ***** |
|  |  |  |  | ctrl-recov | 374.5 | 171.2 | 577.8 | **1.9E-03** | ****** |
|  |  |  |  | heat-recov | 100.0 | -88.3 | 288.2 | 3.3E-01 | ns |
|  |  |  | NP | ctrl-heat | -756.3 | -887.2 | -625.3 | **1.7E-07** | ******** |
|  |  |  |  | ctrl-recov | -1492.5 | -1623.4 | -1361.5 | **1.1E-09** | ******** |
|  |  |  |  | heat-recov | -736.2 | -867.1 | -605.3 | **2.1E-07** | ******** |
|  | Species | ctrl | C | Gal-Heter | -166.5 | -309.5 | -23.5 | **3.0E-02** | ***** |
|  |  |  | NP | Gal-Heter | 2192.6 | 2075.7 | 2309.5 | **4.2E-09** | ******** |
|  |  | heat | C | Gal-Heter | 192.7 | 79.7 | 305.8 | **5.9E-03** | ****** |
|  |  |  | NP | Gal-Heter | -145.3 | -185.6 | -104.9 | **1.2E-04** | ******* |
|  |  | recov | C | Gal-Heter | 1171.7 | 1029.1 | 1314.3 | **9.7E-07** | ******** |
|  |  |  | NP | Gal-Heter | 489.3 | 411.7 | 566.9 | **4.5E-06** | ******** |
|  | **Translocation rate per percentage (Fig. S2f)** | | | | | | | | |
| **3-way ANOVA** | Effect | | | DFn | DFd | F | p | p<.05 | ges |
|  | Species | | | 1 | 35 | 51.57 | **2.24E-08** | ***** | 0.596 |
|  | Temperature | | | 2 | 35 | 142.78 | **1.47E-17** | ***** | 0.891 |
|  | Condition | | | 1 | 35 | 23.99 | **2.18E-05** | ***** | 0.407 |
|  | Species:Temperature | | | 2 | 35 | 119.50 | **2.29E-16** | ***** | 0.872 |
|  | Species:Condition | | | 1 | 35 | 33.94 | **1.31E-06** | ***** | 0.492 |
|  | Temperature:Condition | | | 2 | 35 | 191.44 | **1.42E-19** | ***** | 0.916 |
|  | Species:Temperature:Condition | | | 2 | 35 | 293.93 | **1.32E-22** | ***** | 0.944 |
| **Tukey** | Variable | | | Pairwise comparison | estimate | conf.low | conf.high | p.adj | p.adj.signif |
|  | Nutrient condition | Galaxea | ctrl | C-NP | -76.51 | -85.89 | -67.14 | **1.0E-06** | ******** |
|  |  |  | heat | C-NP | 4.43 | 1.88 | 6.99 | **5.4E-03** | ****** |
|  |  |  | recov | C-NP | 66.20 | 56.64 | 75.76 | **2.6E-06** | ******** |
|  |  | Heterox | ctrl | C-NP | 22.27 | 16.63 | 27.92 | **1.6E-04** | ******* |
|  |  |  | heat | C-NP | 23.75 | 17.48 | 30.03 | **8.9E-05** | ******** |
|  |  |  | recov | C-NP | 2.09 | -9.45 | 13.62 | 6.7E-01 | ns |
|  | Temperature | Galaxea | C | ctrl-heat | 1.09 | -9.26 | 11.44 | 9.5E-01 | ns |
|  |  |  |  | ctrl-recov | -74.39 | -84.75 | -64.04 | **2.2E-08** | ******** |
|  |  |  |  | heat-recov | -75.48 | -85.84 | -65.13 | **1.9E-08** | ******** |
|  |  |  | NP | ctrl-heat | 82.04 | 74.68 | 89.39 | **1.2E-09** | ******** |
|  |  |  |  | ctrl-recov | 68.32 | 60.96 | 75.68 | **2.4E-09** | ******** |
|  |  |  |  | heat-recov | -13.71 | -21.07 | -6.36 | **1.5E-03** | ****** |
|  |  | Heterox | C | ctrl-heat | 12.82 | 0.76 | 24.87 | **3.8E-02** | ***** |
|  |  |  |  | ctrl-recov | 29.53 | 17.48 | 41.59 | **2.9E-04** | ******* |
|  |  |  |  | heat-recov | 16.71 | 5.55 | 27.88 | **6.7E-03** | ****** |
|  |  |  | NP | ctrl-heat | 14.30 | 6.43 | 22.16 | **1.7E-03** | ****** |
|  |  |  |  | ctrl-recov | 9.34 | 1.48 | 17.21 | **2.2E-02** | ***** |
|  |  |  |  | heat-recov | -4.95 | -12.81 | 2.91 | 2.4E-01 | ns |
|  | Species | ctrl | C | Gal-Heter | -38.94 | -50.39 | -27.48 | **3.3E-04** | ******* |
|  |  |  | NP | Gal-Heter | 59.85 | 54.99 | 64.72 | **1.4E-07** | ******** |
|  |  | heat | C | Gal-Heter | -27.21 | -33.61 | -20.81 | **4.6E-05** | ******** |
|  |  |  | NP | Gal-Heter | -7.89 | -10.11 | -5.66 | **1.3E-04** | ******* |
|  |  | recov | C | Gal-Heter | 64.99 | 54.05 | 75.92 | **6.5E-06** | ******** |
|  |  |  | NP | Gal-Heter | 0.87 | -9.37 | 11.12 | 8.4E-01 | ns |
|  | **Carbon loss per AFDW (Fig. S2g)** | | | | | | | | |
| **3-way ANOVA** | Effect | | | DFn | DFd | F | p | p<.05 | ges |
|  | Species | | | 1 | 35 | 1443.07 | **4.82E-30** | ***** | 0.976 |
|  | Temperature | | | 2 | 35 | 618.77 | **4.89E-28** | ***** | 0.972 |
|  | Condition | | | 1 | 35 | 64.05 | **2.04E-09** | ***** | 0.647 |
|  | Species:Temperature | | | 2 | 35 | 240.75 | **3.49E-21** | ***** | 0.932 |
|  | Species:Condition | | | 1 | 35 | 4.27 | **4.60E-02** | ***** | 0.109 |
|  | Temperature:Condition | | | 2 | 35 | 105.75 | **1.46E-15** | ***** | 0.858 |
|  | Species:Temperature:Condition | | | 2 | 35 | 265.65 | **6.97E-22** | ***** | 0.938 |
| **Tukey** | Variable | | | Pairwise comparison | estimate | conf.low | conf.high | p.adj | p.adj.signif |
|  | Nutrient condition | Galaxea | ctrl | C-NP | -317.6 | -431.1 | -204.0 | **4.8E-04** | ******* |
|  |  |  | heat | C-NP | 747.3 | 718.6 | 775.9 | **1.7E-11** | ******** |
|  |  |  | recov | C-NP | -14.5 | -55.3 | 26.3 | 4.2E-01 | ns |
|  |  | Heterox | ctrl | C-NP | 1274.7 | 1097.2 | 1452.2 | **9.5E-06** | ******** |
|  |  |  | heat | C-NP | 54.2 | -112.3 | 220.7 | 4.6E-01 | ns |
|  |  |  | recov | C-NP | -523.4 | -715.6 | -331.2 | **5.5E-04** | ******* |
|  | Temperature | Galaxea | C | ctrl-heat | -66.2 | -166.0 | 33.5 | 2.1E-01 | ns |
|  |  |  |  | ctrl-recov | -745.9 | -845.7 | -646.1 | **1.4E-08** | ******** |
|  |  |  |  | heat-recov | -679.7 | -779.5 | -579.9 | **3.7E-08** | ******** |
|  |  |  | NP | ctrl-heat | 998.6 | 940.3 | 1056.9 | **5.6E-10** | ******** |
|  |  |  |  | ctrl-recov | -442.9 | -501.2 | -384.6 | **1.2E-08** | ******** |
|  |  |  |  | heat-recov | -1441.4 | -1499.7 | -1383.2 | **5.5E-10** | ******** |
|  |  | Heterox | C | ctrl-heat | -64.9 | -330.8 | 201.1 | 7.7E-01 | ns |
|  |  |  |  | ctrl-recov | -229.1 | -495.1 | 36.8 | 9.0E-02 | ns |
|  |  |  |  | heat-recov | -164.2 | -410.5 | 82.0 | 2.0E-01 | ns |
|  |  |  | NP | ctrl-heat | -1285.4 | -1434.9 | -1135.9 | **4.0E-09** | ******** |
|  |  |  |  | ctrl-recov | -2027.2 | -2176.7 | -1877.7 | **7.2E-10** | ******** |
|  |  |  |  | heat-recov | -741.9 | -891.4 | -592.3 | **6.1E-07** | ******** |
|  | Species | ctrl | C | Gal-Heter | 598.1 | 451.3 | 744.9 | **1.4E-04** | ******* |
|  |  |  | NP | Gal-Heter | 2190.3 | 2051.2 | 2329.4 | **2.6E-08** | ******** |
|  |  | heat | C | Gal-Heter | 599.4 | 439.1 | 759.7 | **9.6E-05** | ******** |
|  |  |  | NP | Gal-Heter | -93.6 | -146.9 | -40.4 | **5.1E-03** | ****** |
|  |  | recov | C | Gal-Heter | 1114.9 | 938.5 | 1291.2 | **4.4E-06** | ******** |
|  |  |  | NP | Gal-Heter | 606.0 | 519.4 | 692.5 | **2.4E-06** | ******** |
|  | **Carbon loss per percentage (Fig. S2h)** | | | | | | | | |
| **3-way ANOVA** | Effect | | | DFn | DFd | F | p | p<.05 | ges |
|  | Species | | | 1 | 34 | 0.77 | 0.388 |  | 0.022 |
|  | Temperature | | | 2 | 34 | 1.94 | 0.16 |  | 0.102 |
|  | Condition | | | 1 | 34 | 0.01 | 0.939 |  | 0.000177 |
|  | Species:Temperature | | | 2 | 34 | 5.55 | **0.008** | ***** | 0.246 |
|  | Species:Condition | | | 1 | 34 | 1.26 | 0.269 |  | 0.036 |
|  | Temperature:Condition | | | 2 | 34 | 1.71 | 0.197 |  | 0.091 |
|  | Species:Temperature:Condition | | | 2 | 34 | 0.52 | 0.6 |  | 0.03 |
| **Tukey** | Variable | | | Pairwise comparison | estimate | conf.low | conf.high | p.adj | p.adj.signif |
|  | Nutrient condition | Galaxea | ctrl | C-NP | -2.60 | -9.91 | 4.71 | 0.403 | ns |
|  |  |  | heat | C-NP | 1.70 | -0.44 | 3.84 | 0.100 | ns |
|  |  |  | recov | C-NP | -4.72 | -15.80 | 6.35 | 0.337 | ns |
|  |  | Heterox | ctrl | C-NP | -3.52 | -9.88 | 2.83 | 0.213 | ns |
|  |  |  | heat | C-NP | 5.53 | -3.42 | 14.47 | 0.181 | ns |
|  |  |  | recov | C-NP | 2.07 | -11.56 | 15.70 | 0.723 | ns |
|  | Temperature | Galaxea | C | ctrl-heat | -0.15 | -6.92 | 6.62 | 0.998 | ns |
|  |  |  |  | ctrl-recov | -6.05 | -12.82 | 0.72 | **0.078** | ***** |
|  |  |  |  | heat-recov | -5.89 | -12.16 | 0.37 | **0.064** | ***** |
|  |  |  | NP | ctrl-heat | 4.15 | -6.21 | 14.50 | 0.527 | ns |
|  |  |  |  | ctrl-recov | -8.17 | -18.52 | 2.18 | **0.124** | ***** |
|  |  |  |  | heat-recov | -12.32 | -22.67 | -1.96 | **0.022** | ***** |
|  |  | Heterox | C | ctrl-heat | -7.25 | -22.88 | 8.37 | 0.421 | ns |
|  |  |  |  | ctrl-recov | -2.57 | -18.20 | 13.05 | 0.887 | ns |
|  |  |  |  | heat-recov | 4.68 | -9.78 | 19.14 | 0.641 | ns |
|  |  |  | NP | ctrl-heat | 1.80 | -6.91 | 10.50 | 0.836 | ns |
|  |  |  |  | ctrl-recov | 3.02 | -5.69 | 11.73 | 0.614 | ns |
|  |  |  |  | heat-recov | 1.22 | -7.49 | 9.93 | 0.919 | ns |
|  | Species | ctrl | C | Gal-Heter | -1.76 | -7.11 | 3.60 | 0.414 | ns |
|  |  |  | NP | Gal-Heter | -2.68 | -9.72 | 4.36 | 0.387 | ns |
|  |  | heat | C | Gal-Heter | -8.86 | -17.57 | -0.14 | **0.047** | ***** |
|  |  |  | NP | Gal-Heter | -5.03 | -7.97 | -2.10 | **0.006** | ****** |
|  |  | recov | C | Gal-Heter | 1.72 | -10.77 | 14.20 | 0.748 | ns |
|  |  |  | NP | Gal-Heter | 8.51 | -3.85 | 20.86 | 0.143 | ns |

**Table S3.** **Statistical analysis and results of the effects of nutrient enrichment and warming on coral uptake rates of inorganic nutrients.** “C”, control, non-enriched nubbins; “NP”, nutrient-enriched nubbins; “*ctrl*”, control temperature at 25°C; “heat”, short-term heat stress at 30°C for 14 days; “recov”, recovery phas, with temperature going back to 25°C for 14 days. Significant differences are displayed in bold.

|  | **Uptake rates of ammonium (Fig. 5a)** | | | | | | | |  |
| --- | --- | --- | --- | --- | --- | --- | --- | --- | --- |
| **Kruskal - Wallis** | Grouped by | | variable | | n | statistic | df | p |  |
|  | ctrl | C | Species | | 10 | 0.88 | 1 | 0.347 |  |
|  |  | NP |  |  | 10 | 6.82 | 1 | **0.009** |  |
|  | heat | C |  |  | 10 | 6.82 | 1 | **0.009** |  |
|  |  | NP |  |  | 10 | 1.50 | 1 | 0.221 |  |
|  | recov | C |  |  | 10 | 6.82 | 1 | **0.009** |  |
|  |  | NP |  |  | 10 | 3.15 | 1 | 0.076 |  |
|  | Galaxea | C | Temperature | | 15 | 2.34 | 2 | 0.310 |  |
|  |  | NP |  |  | 15 | 1.83 | 2 | 0.401 |  |
|  | Heterox | C |  |  | 15 | 10.50 | 2 | **0.005** |  |
|  |  | NP |  |  | 15 | 10.82 | 2 | **0.004** |  |
|  | Galaxea | ctrl | Nutrient condition | | 10 | 0.24 | 1 | 0.624 |  |
|  |  | heat |  |  | 10 | 0.01 | 1 | 0.917 |  |
|  |  | recov |  |  | 10 | 0.53 | 1 | 0.465 |  |
|  | Heterox | ctrl |  |  | 10 | 0.53 | 1 | 0.465 |  |
|  |  | heat |  |  | 10 | 6.82 | 1 | **0.009** |  |
|  |  | recov |  |  | 10 | 0.53 | 1 | 0.465 |  |
| **Dunn** |  | | | Pairwise comparison | statistic | p | p.adj | p.adj.signif |  |
|  | Species | ctrl | C | Gal-Heter | -0.94 | 0.347 | 0.347 | ns |  |
|  |  |  | NP | Gal-Heter | -2.61 | 0.009 | **0.009** | ****** |  |
|  |  | heat | C | Gal-Heter | -2.61 | 0.009 | **0.009** | ****** |  |
|  |  |  | NP | Gal-Heter | -1.22 | 0.221 | 0.221 | ns |  |
|  |  | recov | C | Gal-Heter | 2.61 | 0.009 | **0.009** | ****** |  |
|  |  |  | NP | Gal-Heter | -1.78 | 0.076 | 0.076 | ns |  |
|  | Temperature | Galaxea | C | ctrl-heat | 1.06 | 0.289 | 0.578 | ns |  |
|  |  |  |  | ctrl-recov | 1.48 | 0.138 | 0.413 | ns |  |
|  |  |  |  | heat-recov | 0.42 | 0.671 | 0.671 | ns |  |
|  |  |  | NP | ctrl-heat | 1.34 | 0.181 | 0.544 | ns |  |
|  |  |  |  | ctrl-recov | 0.91 | 0.364 | 0.727 | ns |  |
|  |  |  |  | heat-recov | -0.45 | 0.650 | 0.727 | ns |  |
|  |  | Heterox | C | ctrl-heat | -2.12 | 0.034 | **0.048** | ***** |  |
|  |  |  |  | ctrl-recov | -3.18 | 0.001 | **0.004** | ****** |  |
|  |  |  |  | heat-recov | -1.06 | 0.289 | 0.289 | ns |  |
|  |  |  | NP | ctrl-heat | 2.05 | 0.040 | **0.006** | ****** |  |
|  |  |  |  | ctrl-recov | -1.20 | 0.229 | 0.229 | ns |  |
|  |  |  |  | heat-recov | -3.25 | 0.001 | **0.003** | ****** |  |
|  | Nutrient condition | Galaxea | ctrl | C-NP | -0.49 | 0.624 | 0.624 | ns |  |
|  |  |  | heat | C-NP | -0.10 | 0.917 | 0.917 | ns |  |
|  |  |  | recov | C-NP | -0.73 | 0.465 | 0.465 | ns |  |
|  |  | Heterox | ctrl | C-NP | -0.73 | 0.465 | 0.465 | ns |  |
|  |  |  | heat | C-NP | 2.61 | 0.009 | **0.009** | ****** |  |
|  |  |  | recov | C-NP | 0.73 | 0.465 | 0.465 | ns |  |
|  | **Uptake rates of nitrate (Fig. 5b)** | | | | | | | | |
| **3-way ANOVA** | Effect | | | DFn | DFd | F | p | p<.05 | ges |
|  | Species | | | 1 | 44 | 0.28 | 5.99E-01 |  | 0.006 |
|  | Temperature | | | 2 | 44 | 15.60 | **7.56E-06** | ***** | 0.415 |
|  | Condition | | | 1 | 44 | 22.62 | **2.15E-05** | ***** | 0.34 |
|  | Species:Temperature | | | 2 | 44 | 4.20 | **2.10E-02** | ***** | 0.16 |
|  | Species:Condition | | | 1 | 44 | 2.86 | 9.80E-02 |  | 0.061 |
|  | Temperature:Condition | | | 2 | 44 | 4.14 | **2.20E-02** | ***** | 0.158 |
|  | Species:Temperature:Condition | | | 2 | 44 | 6.51 | **3.00E-03** | ***** | 0.228 |
| **Tukey** | Variable | | | Pairwise comparison | estimate | conf.low | conf.high | p.adj | p.adj.signif |
|  | Nutrient condition | Galaxea | ctrl | C-NP | 20.24 | 11.68 | 28.81 | **0.001** | ******* |
|  |  |  | heat | C-NP | -4.44 | -14.06 | 5.19 | 0.312 | ns |
|  |  |  | recov | C-NP | 5.19 | -0.29 | 10.68 | 0.060 | ns |
|  |  | Heterox | ctrl | C-NP | 16.89 | 0.44 | 33.34 | **0.046** | ***** |
|  |  |  | heat | C-NP | 26.33 | 4.06 | 48.61 | **0.026** | ***** |
|  |  |  | recov | C-NP | 0.41 | -2.48 | 3.30 | 0.753 | ns |
|  | Temperature | Galaxea | C | ctrl-heat | 20.37 | 9.67 | 31.06 | **0.001** | ******* |
|  |  |  |  | ctrl-recov | 16.41 | 5.06 | 27.75 | **0.006** | ****** |
|  |  |  |  | heat-recov | -3.96 | -15.30 | 7.39 | 0.626 | ns |
|  |  |  | NP | ctrl-heat | -4.31 | -10.87 | 2.24 | 0.217 | ns |
|  |  |  |  | ctrl-recov | 1.36 | -4.86 | 7.57 | 0.824 | ns |
|  |  |  |  | heat-recov | 5.67 | -0.54 | 11.89 | 0.074 | ns |
|  |  | Heterox | C | ctrl-heat | 16.90 | 1.85 | 31.96 | **0.028** | ***** |
|  |  |  |  | ctrl-recov | 15.58 | 0.53 | 30.64 | **0.043** | ***** |
|  |  |  |  | heat-recov | -1.32 | -15.51 | 12.88 | 0.966 | ns |
|  |  |  | NP | ctrl-heat | 26.34 | 5.17 | 47.52 | **0.016** | ***** |
|  |  |  |  | ctrl-recov | -0.90 | -22.08 | 20.28 | 0.993 | ns |
|  |  |  |  | heat-recov | -27.24 | -48.42 | -6.07 | **0.013** | ***** |
|  | Species | ctrl | C | Gal-Heter | -3.28 | -20.64 | 14.08 | 0.669 | ns |
|  |  |  | NP | Gal-Heter | -6.63 | -13.17 | -0.09 | **0.048** | ***** |
|  |  | heat | C | Gal-Heter | -6.74 | -15.72 | 2.23 | 0.121 | ns |
|  |  |  | NP | Gal-Heter | 24.03 | -1.55 | 49.61 | 0.062 | ns |
|  |  | recov | C | Gal-Heter | -4.11 | -8.33 | 0.12 | 0.055 | ns |
|  |  |  | NP | Gal-Heter | -8.89 | -13.06 | -4.72 | **0.001** | ****** |
|  | **Uptake rates of phosphate (Fig. 5c)** | | | | | | | | |
| **3-way ANOVA** | Effect | | | DFn | DFd | F | p | p<.05 | ges |
|  | Species | | | 1 | 42 | 0.06 | 8.06E-01 |  | 0.001 |
|  | Temperature | | | 2 | 42 | 22.46 | **2.33E-07** | ***** | 0.517 |
|  | Condition | | | 1 | 42 | 0.01 | 9.28E-01 |  | 0.000195 |
|  | Species:Temperature | | | 2 | 42 | 7.00 | **2.00E-03** | ***** | 0.25 |
|  | Species:Condition | | | 1 | 42 | 18.67 | **9.33E-05** | ***** | 0.308 |
|  | Temperature:Condition | | | 2 | 42 | 2.44 | 9.90E-02 |  | 0.104 |
|  | Species:Temperature:Condition | | | 2 | 42 | 14.52 | **1.61E-05** | ***** | 0.409 |
| **Tukey** | Variable | | | Pairwise comparison | estimate | conf.low | conf.high | p.adj | p.adj.signif |
|  | Nutrient condition | Galaxea | ctrl | C-NP | -1.02 | -2.01 | -0.03 | **0.046** | ***** |
|  |  |  | heat | C-NP | -2.24 | -3.16 | -1.32 | **4.90E-04** | ******* |
|  |  |  | recov | C-NP | 0.23 | -2.61 | 3.06 | 0.846 | ns |
|  |  | Heterox | ctrl | C-NP | -0.33 | -0.88 | 0.23 | 0.199 | ns |
|  |  |  | heat | C-NP | 3.66 | 1.26 | 6.07 | **0.008** | ****** |
|  |  |  | recov | C-NP | -0.28 | -0.59 | 0.03 | 0.067 | ns |
|  | Temperature | Galaxea | C | ctrl-heat | 1.10 | -0.08 | 2.29 | 0.068 | ns |
|  |  |  |  | ctrl-recov | -0.90 | -2.16 | 0.35 | 0.173 | ns |
|  |  |  |  | heat-recov | -2.01 | -3.26 | -0.75 | **0.003** | ****** |
|  |  |  | NP | ctrl-heat | -0.12 | -2.20 | 1.96 | 0.985 | ns |
|  |  |  |  | ctrl-recov | 0.34 | -2.02 | 2.70 | 0.916 | ns |
|  |  |  |  | heat-recov | 0.46 | -1.80 | 2.72 | 0.839 | ns |
|  |  | Heterox | C | ctrl-heat | 0.38 | -0.20 | 0.96 | 0.229 | ns |
|  |  |  |  | ctrl-recov | -0.75 | -1.33 | -0.17 | **0.013** | ***** |
|  |  |  |  | heat-recov | -1.13 | -1.67 | -0.58 | **4.58E-04** | ******* |
|  |  |  | NP | ctrl-heat | 4.37 | 1.83 | 6.90 | **0.002** | ****** |
|  |  |  |  | ctrl-recov | -0.70 | -3.24 | 1.83 | 0.741 | ns |
|  |  |  |  | heat-recov | -5.07 | -7.46 | -2.68 | **3.60E-04** | ******* |
|  | Species | ctrl | C | Gal-Heter | -0.98 | -1.86 | -0.10 | **0.033** | ***** |
|  |  |  | NP | Gal-Heter | -0.29 | -1.07 | 0.48 | 0.394 | ns |
|  |  | heat | C | Gal-Heter | -1.71 | -2.59 | -0.82 | **0.002** | ****** |
|  |  |  | NP | Gal-Heter | 4.20 | 1.78 | 6.61 | **0.004** | ****** |
|  |  | recov | C | Gal-Heter | -0.83 | -1.51 | -0.14 | **0.025** | ***** |
|  |  |  | NP | Gal-Heter | -1.33 | -3.58 | 0.91 | 0.196 | ns |

**Table S4.** **Summary of results expressed as mean** ± **sd.**

| PHOTOPHYSIOLOGY | | | | | | |
| --- | --- | --- | --- | --- | --- | --- |
| **Species** | **Temperature** | **Condition** | **Symbiont density** | **Chl/symbiont** | **Chl/AFDW** | **P_R** |
| Galaxea | ctrl | C | 149388 ± 87244 | 13.12 ± 3.52 | 0.2 ± 0.13 | 1.29 ± 0.06 |
| Galaxea | ctrl | NP | 96872 ± 46450 | 10.69 ± 1.07 | 0.16 ± 0.1 | 0.9 ± 0.14 |
| Galaxea | heat | C | 119937 ± 82999 | 6.22 ± 1.15 | 0.07 ± 0.06 | 0.81 ± 0.07 |
| Galaxea | heat | NP | 136663 ± 30460 | 5.69 ± 2.04 | 0.07 ± 0.01 | 1.25 ± 0.08 |
| Galaxea | post stress | C | 20946 ± 11825 | 34.53 ± 11.91 | 0.09 ± 0.04 | 1.05 ± 0.34 |
| Galaxea | post stress | NP | 46487 ± 12330 | 17.63 ± 6.03 | 0.08 ± 0.04 | 1.29 ± 0.73 |
| Heteroxenia | ctrl | C | 826641 ± 497565 | 11.25 ± 3.01 | 0.87 ± 0.39 | 1 ± 0.16 |
| Heteroxenia | ctrl | NP | 1387112 ± 652316 | 9.88 ± 2.54 | 1.37 ± 0.75 | 0.94 ± 0.15 |
| Heteroxenia | heat | C | 1024491 ± 292159 | 10.13 ± 2.14 | 1.03 ± 0.33 | 0.9 ± 0.14 |
| Heteroxenia | heat | NP | 383480 ± 146414 | 5.27 ± 0.76 | 0.21 ± 0.12 | 1.24 ± 0.22 |
| Heteroxenia | post stress | C | 1271750 ± 145114 | 16.82 ± 1.51 | 1.96 ± 0.35 | 0.74 ± 0.16 |
| Heteroxenia | post stress | NP | 462567 ± 322893 | 12.38 ± 2.73 | 0.52 ± 0.28 | 1.08 ± 0.19 |
| UPTAKE OF INORGANIC NUTRIENTS | | | | | | |
| **Species** | **Temperature** | **Condition** | **NH4** | **NO3** | **PO4** |  |
| Galaxea | ctrl | C | 6.73 ± 3.81 | -7.77 ± 6.52 | 2.03 ± 0.68 |  |
| Galaxea | ctrl | NP | 5.27 ± 2.76 | 12.47 ± 3.37 | 1.02 ± 0.54 |  |
| Galaxea | heat | C | 10.71 ± 4.51 | 12.59 ± 7.78 | 3.14 ± 0.78 |  |
| Galaxea | heat | NP | 10.68 ± 7.13 | 8.16 ± 2.28 | 0.89 ± 0.43 |  |
| Galaxea | post stress | C | 12.50 ± 7.78 | 8.64 ± 2.54 | 1.13 ± 0.58 |  |
| Galaxea | post stress | NP | 9.90 ± 7.89 | 13.83 ± 4.01 | 1.36 ± 1.77 |  |
| Heteroxenia | ctrl | C | 3.84 ± 0.62 | -11.05 ± 14.93 | 1.05 ± 0.32 |  |
| Heteroxenia | ctrl | NP | 3.40 ± 1.45 | 5.84 ± 4.60 | 0.73 ± 0.32 |  |
| Heteroxenia | heat | C | 2.02 ± 0.80 | 5.85 ± 3.90 | 1.43 ± 0.36 |  |
| Heteroxenia | heat | NP | 46.82 ± 19.87 | 32.18 ± 21.24 | 5.09 ± 2.30 |  |
| Heteroxenia | post stress | C | 1.07 ± 1.18 | 4.53 ± 2.76 | 0.30 ± 0.27 |  |
| Heteroxenia | post stress | NP | 1.79 ± 0.80 | 4.94 ± 0.47 | 0.02 ± 0.12 |  |
| ENERGETIC RESERVES | | | | | | |
| **Species** | **Temperature** | **Condition** | **Proteins** | **Lipids** | **Carbohydrates** | **Total energy** |
| Galaxea | ctrl | C | 164.59 ± 62.58 | 69.09 ± 19.63 | 51.12 ± 22.47 | 6.83 ± 2.14 |
| Galaxea | ctrl | NP | 237.18 ± 39.90 | 92.79 ± 7.28 | 56.11 ± 25.32 | 9.16 ± 0.77 |
| Galaxea | heat | C | 214.11 ± 43.62 | 89.84 ± 22.93 | 38.46 ± 19.50 | 8.21 ± 1.63 |
| Galaxea | heat | NP | 190.16 ± 18.74 | 84.66 ± 7.48 | 89.09 ± 6.45 | 9.11 ± 1.10 |
| Galaxea | post stress | C | 217.59 ± 67.19 | 112.58 ± 5.54 | 168.88 ± 42.55 | 12.29 ± 2.23 |
| Galaxea | post stress | NP | 199.53 ± 20.13 | 134.05 ± 51.90 | 87.20 ± 37.93 | 10.45 ± 2.48 |
| Heteroxenia | ctrl | C | 201.09 ± 19.80 | 170.68 ± 12.65 | 93.46 ± 35.72 | 12.49 ± 0.92 |
| Heteroxenia | ctrl | NP | 205.00 ± 27.40 | 196.60 ± 7.71 | 88.78 ± 22.60 | 13.12 ± 0.71 |
| Heteroxenia | heat | C | 206.92 ± 21.00 | 196.43 ± 19.79 | 68.69 ± 12.04 | 13.02 ± 0.91 |
| Heteroxenia | heat | NP | 153.99 ± 20.85 | 226.77 ± 23.82 | 57.69 ± 14.31 | 13.35 ± 1.32 |
| Heteroxenia | post stress | C | 212.55 ± 20.38 | 303.70 ± 56.24 | 136.84 ± 22.42 | 18.66 ± 2.43 |
| Heteroxenia | post stress | NP | 186.01 ± 27.48 | 438.72 ± 43.56 | 149.99 ± 11.83 | 24.17 ± 2.22 |
